# FOXC2 represses NFAT1-dependent transcription through a DNA-facilitated protein–protein interaction

**DOI:** 10.1093/nar/gkag367

**Published:** 2026-04-23

**Authors:** Xiaojuan Chen, Sipeng Wu, Sitong Yue, Lin Zhang, Xueru Liu, Shuyan Dai, Jun Li, Huajun Zhang, Hudie Wei, Ming Guo, Lingzhi Qu, Lin Chen, Yalan Deng, Yongheng Chen

**Affiliations:** Department of Oncology, NHC Key Laboratory of Cancer Proteomics & State Local Joint Engineering Laboratory for Anticancer Drugs, National Clinical Research Center for Geriatric Diseases, Xiangya Hospital, Central South University, Changsha, Hunan 410008, China; Department of Gastroenterology, Xiangya Hospital, Central South University, Changsha 410008 Hunan, China; Department of Ultrasound, Xiangyang No. 1 People’s Hospital, Hubei University of Medicine, Xiangyang 441000, China; Department of Oncology, NHC Key Laboratory of Cancer Proteomics & State Local Joint Engineering Laboratory for Anticancer Drugs, National Clinical Research Center for Geriatric Diseases, Xiangya Hospital, Central South University, Changsha, Hunan 410008, China; Department of Oncology, NHC Key Laboratory of Cancer Proteomics & State Local Joint Engineering Laboratory for Anticancer Drugs, National Clinical Research Center for Geriatric Diseases, Xiangya Hospital, Central South University, Changsha, Hunan 410008, China; Department of Oncology, NHC Key Laboratory of Cancer Proteomics & State Local Joint Engineering Laboratory for Anticancer Drugs, National Clinical Research Center for Geriatric Diseases, Xiangya Hospital, Central South University, Changsha, Hunan 410008, China; Department of Pharmacology, Xiangya School of Pharmaceutical Sciences, Central South University, Changsha 410078, China; Institute of Clinical Medicine, The First Affiliated Hospital of University of South China, Hengyang, Hunan 421001, China; Department of Oncology, NHC Key Laboratory of Cancer Proteomics & State Local Joint Engineering Laboratory for Anticancer Drugs, National Clinical Research Center for Geriatric Diseases, Xiangya Hospital, Central South University, Changsha, Hunan 410008, China; Department of Ultrasonic Imaging, Xiangya Hospital, Central South University, Changsha 410008 Hunan, China; Department of Oncology, NHC Key Laboratory of Cancer Proteomics & State Local Joint Engineering Laboratory for Anticancer Drugs, National Clinical Research Center for Geriatric Diseases, Xiangya Hospital, Central South University, Changsha, Hunan 410008, China; Department of Oncology, NHC Key Laboratory of Cancer Proteomics & State Local Joint Engineering Laboratory for Anticancer Drugs, National Clinical Research Center for Geriatric Diseases, Xiangya Hospital, Central South University, Changsha, Hunan 410008, China; Department of Oncology, NHC Key Laboratory of Cancer Proteomics & State Local Joint Engineering Laboratory for Anticancer Drugs, National Clinical Research Center for Geriatric Diseases, Xiangya Hospital, Central South University, Changsha, Hunan 410008, China; Molecular and Computational Biology, Department of Biological Sciences, University of Southern California, Los Angeles, CA 90089, United States; Department of Oncology, NHC Key Laboratory of Cancer Proteomics & State Local Joint Engineering Laboratory for Anticancer Drugs, National Clinical Research Center for Geriatric Diseases, Xiangya Hospital, Central South University, Changsha, Hunan 410008, China; Department of Ultrasonic Imaging, Xiangya Hospital, Central South University, Changsha 410008 Hunan, China; Department of Oncology, NHC Key Laboratory of Cancer Proteomics & State Local Joint Engineering Laboratory for Anticancer Drugs, National Clinical Research Center for Geriatric Diseases, Xiangya Hospital, Central South University, Changsha, Hunan 410008, China

## Abstract

Transcription factor nuclear factor of activated T cells (NFAT) plays a central role in immune gene regulation through cooperative interactions with diverse transcriptional partners. While FOXP family members have been identified as co-regulators of NFAT1, the involvement of other FOX family proteins has remained mechanistically obscure. Here, we solved three crystal structures of NFAT1–RHR/FOXC2–DBD/ARRE DNA ternary complexes and uncovered an unexpected mode of transcriptional repression mediated by FOXC2 through direct, DNA-facilitated binding to the V-shaped groove of NFAT1’s Rel-homology region (RHR). Biochemical assays revealed that DNA enhanced FOXC2–NFAT1 interaction by more than five-fold, supporting a model in which DNA acts as a structural co-factor that promotes complex formation. Mutational disruption of the FOXC2–NFAT1 interface impaired complex assembly and abrogated transcriptional repression. Functional assays further confirmed that FOXC2 suppressed NFAT1-driven transcription of multiple cytokines and chemokines, including IL2, TNF, CXCL5, and CCL2. Notably, this repressive mechanism was found to extend to other FOX proteins (FOXI1, FOXO1, and FOXK1), suggesting a broader paradigm of FOX–NFAT1 interaction. Our study defined a previously unrecognized FOX-mediated transcriptional repression mechanism and provides a structural framework for NFAT inhibition by FOX proteins, offering novel insights into the transcriptional regulation of immune-related genes.

## Graphical Abstract

**Graphical Abstract:**
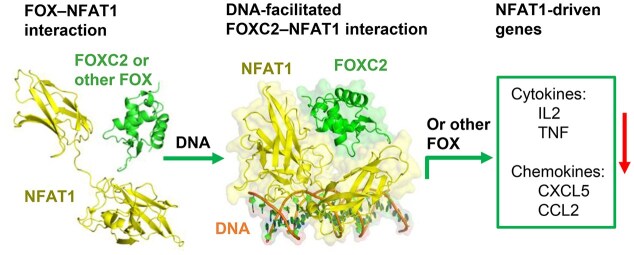

## Introduction

The nuclear factor of activated T cells (NFAT) family consists of five calcium-sensitive transcription factors: NFAT1 (NFATc2/NFATp), NFAT2 (NFATc1/NFATc), NFAT3 (NFATc4), NFAT4 (NFATc3/NFATx), and NFAT5 (TonEBP) [1]. NFAT1 plays a central role in regulating gene expression in diverse biological processes, including those in the immune system, as well as in tissues such as muscle, bone, and brain [2–4]. Structurally, NFAT1 is composed of two functionally distinct domains: an N-terminal regulatory domain and a Rel-homology region (RHR), the latter of which mediates both DNA binding and partner interactions [4, 5]. Upon dephosphorylation by calcineurin, NFAT1 translocated into the nucleus and binds to composite DNA elements such as ARRE (antigen receptor response element) to regulate the transcription of cytokines and chemokines, notably IL2, TNF, and CCL2 [6–9].

Members of the forkhead box (FOX) family of transcription factors have been reported as important modulators of NFAT-mediated gene expression. Among them, FOXP1, FOXP2, and FOXP3 physically interact with NFAT1 and act as either co-repressors or co-activators in a DNA-dependent manner by binding specific motifs adjacent to NFAT-binding sites [10–12]. For example, FOXP3 is critical for regulatory T-cell development and mediates transcriptional repression by forming stable complexes with NFAT1 on regulatory elements [13–16]. Notably, recent studies have shown that FOX family members beyond the FOXP subfamily can also regulate NFAT activity. FOXD3, a pluripotency-associated transcription factor, interacts with NFAT proteins and suppress NFATc3-driven transcriptional programs, thereby maintaining embryonic stem cell identity [17]. Collectively, these findings suggest that FOX proteins, both within and beyond the FOXP subfamily, play more extensive roles in modulating NFAT signaling across different cellular contexts. However, whether additional FOX members participate in NFAT1 regulation remains largely unknown.

FOXC2 is a distinct FOX family transcription factor involved in vascular development, epithelial–mesenchymal transition, and tumor progression [18, 19]. Unlike FOXP proteins, FOXC2 has not been reported to directly interact with NFAT1 or act as a transcriptional co-repressor. Intriguingly, genome-wide data suggest that FOXC2 and NFAT1 frequently co-localize at regulatory regions of genes, raising the possibility of functional interaction [20]. FOXC2 has been shown to cooperate with NFATc1 controlling the maturation of collecting lymphatic vessels and the formation of venous valve, as well as promoting the growth of leaflets at later developmental stages [20, 21]. In addition, FOXC2, in conjunction with PROX1 and fluid shear stress, regulates lymphatic valve formation by modulating Connexin37 expression and calcineurin/NFAT signaling pathway [22]. However, the mechanism by which FOXC2 might influence NFAT1-dependent transcription remains unclear, particularly considering the absence of canonical FOX–NFAT motifs and FOXC2’s structural divergence from FOXP subfamily members.

In this study, we report a previously uncharacterized mechanism of transcriptional repression in which FOXC2 directly engages NFAT1 through a DNA-facilitated protein–protein interaction. Using crystallographic, biochemical, and cellular approaches, we demonstrate that FOXC2 acts as a co-repressor of NFAT1 and suppresses the transcription of multiple cytokines and chemokines. Moreover, we reveal that this repression mechanism extends to other FOX proteins, indicating a broader FOX–NFAT1 regulatory mechanism. These findings provide new mechanistic insights into immune gene regulation and expand the functional repertoire of the FOX family.

## Materials and methods

### Plasmids

Human NFAT1 (amino acids 392–678), NFAT2 (amino acids 409–696), NFAT3 (amino acids 401–686), and NFAT4 (amino acids 415–700) were cloned into pET-28a with an N-terminal 6 × His-tag. Human FOXC2 (amino acids 72–172), FOXP3–DBD (amino acids 336–420), FOXK1 (amino acids 302–400), FOXI1 (amino acids 120–223), and FOXO1 (amino acids 151–265) were cloned into pGEX-6p-1 with an N-terminal GST-tag and pET-28a with an N-terminal 6 × His-tag followed by a PreScission protease cleavage site, respectively.

Full-length NFAT1 and FOXP3 used in cellular assays was cloned into the mammalian expression vector pcDNA3.1 with a C-terminal 3 × Flag-tag. Full-length FOXC2, FOXO1, FOXI1, and FOXK1 were cloned into pcDNA3.1 vector with a C-terminal HA-tag. Wild-type and mutated human *IL2* promoter element ARRE2 DNAs with three repeats for luciferase reporter gene assay were synthesized and subcloned into the firefly luciferase reporter vector pGL4.27.

All plasmids were constructed through homologous recombination technology according to the manufacturer’s instructions for ClonExpress II One Step Cloning Kit (Vazyme, C112). All site-directed mutants were performed using the wild-type plasmid as the template and obtained with the KOD-Plus-Mutagenesis kit (TOYOBO, SMK-101). All plasmids were confirmed by DNA sequencing (Tsingke).

### Protein expression and purification

NFAT and FOX family proteins were recombinantly expressed in *Escherchia coli* BL21 (DE3) cells (Tsingke, TSC-E01). The cells were first grown at 37°C in LB culture when the culture OD600 reached 0.8. Then the cells were induced with 0.5 mM isopropyl β-d-1-thiogalactoside (IPTG) and further incubated for 20 h at 30°C (NFAT proteins) or 20°C (FOX proteins). Cells were harvested by centrifugation at 3000 × *g* for 30 min. For purification, cells were resuspended in lysis buffer (50 mM HEPES pH 7.5, 500 mM NaCl, 10 mM phenylmethylsulfonyl fluoride (PMSF), and 5 mM β-ME) and lysed with a high-pressure homogenizer. The cell lysate was further clarified by centrifugation at 18 000 × *g* for 30 min. Then, the supernatant was subjected to purification.

His-tagged NFAT and FOX family proteins were purified by nickel affinity chromatography (GE Healthcare, 17-5318-02), followed by cation-exchange chromatography (GE Healthcare, 17-5168-01). Untagged FOX was obtained by incubation with PreScission protease at 4°C before cation-exchange chromatography. GST fusion proteins of FOX were purified by glutathione sepharose (GE Healthcare, 17-0756-01) and cation-exchange chromatography. For crystallization, His-NFAT1 and FOXC2 were further purified by gel filtration chromatography Superdex 7510/300 GL (GE Healthcare, 29-1487-21). Purified NFAT proteins were concentrated to 0.8–2 mM in 10 mM HEPES pH 7.5, 100 mM NaCl, 0.5 M NH_4_OAc, 1 mM TCEP buffer, and purified FOX proteins were concentrated to 0.5–1.5 mM in 20 mM HEPES pH 7.5, 200 mM NaCl, and 1 mM TCEP buffer. Proteins were flash frozen in liquid nitrogen, and stored at −80°C for further usage.

### Duplex DNA preparation

All single-stranded DNA oligonucleotides were purchased from Genewiz (SuZhou, China). Duplex DNAs were annealed and purified as described previously reports [16, 23]. The human *IL2* promoter element ARRE2 (hARRE2) contains strands 5′-TTAGGAAAAACTGTTTCATAG-3′ and 5′-AACTATGAAACAGTTTTTCCT-3′, and the mouse *IL2* promoter element ARRE2 (mARRE2) contains strands 5′-TTAGGAAAATTTGTTTCATAG-3′ and 5′-AACTATGAAACAAATTTTCCT-3′ [10]. The blunt end mARRE2 (ARRE2-P) sites: 5′-TTAGGAAAATTTGTTTCATAG-3′and 5′-CTATGAAACAAATTTTCCTAA-3′; modified cohesive end ARRE2 (ARRE3) sites: 5′-TTAGGAAAATTTGTTTACTAG-3′ and 5′-AACTAGTAAACAAATTTTCCT-3′; modified blunt end ARRE2 (ARRE3-P) sites: 5′-TTAGGAAAATTTGTTTACTAG-3′and 5′-CTAGTAAACAAATTTTCCTAA-3′. The control DBE2 contains strands 5′-CAAAATGTAAACAAGA-3′ and 5′-TCTTGTTTACATTTTG-3′ [24, 25]. All DNAs used for crystallization, electrophoretic mobility shift assays (EMSAs) and surface plasmon resonance (SPR) were annealed to 1000, 45, and 45 μM, respectively. For SPR experiments specifically, the corresponding sense strands were 5′-biotinylated, and the duplexes were annealed using the same protocol to obtain biotinylated DNA probes.

### Crystallization

NFAT1−FOXC2−DNA complexes were prepared by mixing NFAT1, FOXC2, and DNA at 1:1.5:1.2 molar ratio with a final protein concentration of 10 mg/ml. Crystal screening was performed by hanging drop vapor diffusion in a 24-well plate by mixing 0.8 μl of protein solution and 0.8 μl of mother liquor. The mixture was equilibrated against 500 μl of the reservoir at 4°C. Crystals appeared in some drops after a week, and then optimizations were carried out by hanging drop vapour diffusion at 4°C. The optimal crystals of FOXC2/NFAT/DNA complexes appeared in reservoir buffer containing 50 mM MES pH 5.93, 200 mM NaCl, 10 mM MgCl_2_, 1 mM TCEP, and 12%–15% PEG 4K (w/v). Then, crystals were soaked in a well solution harboring 20% glycerol (v/v) and flash-frozen in liquid nitrogen for cryo-crystallography.

### EMSA

Binding reactions were performed in a total volume of 6 μl in 20 mM HEPES pH 7.5, 200 mM NaCl, 10 mM MgCl2, 0.5 mM EDTA, 1 mM TCEP, 0.2%Triton X 100, and 15% glycerol. NFAT proteins, FOX proteins, and DNAs were diluted to about 45 μM and mutually incubated at room temperature for 20 minutes. Free DNAs were separated from the proteins/DNAs complex with 8% (w/v) polyacrylamide gel (PAGE) in 0.5× TBE buffer. The gel could be observed by GelBlu™ (UE, S2019L) staining.

### Data collection and structure determination

Data sets of FOXC2/NFAT/DNA complexes were collected at beamlines BL19U1 [26] of the Shanghai Synchrotron Radiation Facility (SSRF). HKL2000 was used to index and process the data [27]. Structures were determined by molecular replacement (MR) using Phenix.phaser [28]. The initial model of FOXC2/NFAT/ARRE2 ternary complex was first determined by using the NFAT1 structure (PDB: 1A02) [6], FOXC2 structure (PDB: 6AKO) [23], and ARRE2 structure (PDB: 2AS5) [10] as three separate search templates. The further model was manually built by the program Coot and refinement was performed with Phenix.refine. Then, FOXC2/NFAT/ARRE2-P and FOXC2/NFAT/ARRE3-P crystal structures were resolved by MR using FOXC2/NFAT/ARRE2 as the search model. The statistics of data collection and structure refinement are summarized in Supplementary Table S1. All structural figures were prepared using PyMOL.

### Pull-down

20 μM GST or GST-FOX proteins were incubated with 20 μM His-NFAT proteins for 2 h at 4°C in 50 μl of PBS buffer containing 1 mM TCEP. Then, 20 μl of Nickel or Glutathione-Sepharose beads were added to the mixture at 4°C for 10 min. The samples were centrifuged at 500 × *g* for 5 min at 4°C in a microcentrifuge. Beads were further washed 3–5 times with PBS buffer containing 1 mM TCEP and 0.5% Triton X-100. 50 mM imidazole was added to the same buffer for Ni-NTA pull-down. Finally, the samples were resuspended with 100 μl of 1× SDS–PAGE loading buffer and boiled in a water bath for 10 min before western blotting detection.

### Isothermal titration calorimetry (ITC)

Titration experiments were performed with a Nano ITC instrument (TA) in 20 mM HEPES pH 7.5, 50 mM NaCl, and 1 mM TCEP at 20°C. NFAT1 protein was pre-incubated with ARRE2 DNA at a 1:1 molar ratio for 30 min at room temperature prior to titrations. FOXC2 protein was titrated into either NFAT1 alone or pre-complexed NFAT1–ARRE2. The titration consisted of 25 injections of 2 μl each at 240 s intervals between injections. Data were fitted using an independent model to obtain dissociation constant (*K*_d_), binding enthalpy (Δ*H*), and stoichiometry (*n*) using NanoAnalyze Software.

### Surface plasmon resonance assay

The SPR assays were performed using a Biacore 8K system (Cytiva) at 25°C in HBS-EP running buffer (Cytiva, BR100669). For protein–protein interaction analysis, carboxyl groups on a Series S CM5 sensor chip (Cytiva, 29104988) were activated with a 1:1 mixture of *N*-hydroxysuccinimide (NHS) and *N*-ethyl-N′-(3-dimethylaminopropyl) carbodiimide (EDC) to form active esters. FOXC2 or NFAT protein was then immobilized via standard amine-coupling chemistry to a density of approximately 1000 response units (RU). Binding assays were performed by injecting diluted solutions of the analytes (e.g. NFAT or FOX proteins, or a pre-incubated mixture of FOXC2 and a fixed DNA concentration) in HBS-EP buffer at a flow rate of 30 µl/min, with contact times of 90 s and dissociation times of 150–300 s. The sensor surface was regenerated after each cycle with 10 mM glycine–HCl, pH 1.5. Equilibrium dissociation constants were calculated using two state reaction model, and data were plotted using GraphPad Prism software.

For DNA–protein interaction studies, a Series S SA sensor chip (Cytiva, 29104992) was used. The SA chip was first activated with a condition buffer containing 50 mM NaOH and 1 M NaCl, then biotinylated double-stranded DNAs were immobilized on the SA chip surface by injection at 10 µl/min in HBS-EP buffer until a capture level of approximately 300–500 RU was achieved. Protein samples or a pre-incubated mixture of NFAT1–FOX were then injected over the DNA-immobilized surface under the same buffer and flow conditions as described above. The sensor chip was regenerated with 0.5% SDS. Binding constants were also derived from two state reaction fitting using the Biacore 8K evaluation software.

### Co-immunoprecipitation (Co-IP)

HEK 293T cells were seeded into 10 cm^2^ dishes to reach 50%–70% confluence. The cells were transfected with pcDNA3.1–NFAT1 and pcDNA3.1–FOXC2 plasmids for 48 h using jetPRIME (Polyplus, 101000046) according to the manufacturer’s instructions. The total protein of HEK 293T cells was lysed by 1% Triton X-100 (Sigma–Aldrich) buffer supplemented with protease inhibitor cocktail (Roche, 11697498001). Then, the mixtures were incubated with FLAG conjugated agarose beads (Sino Biological, 101274-MM13-RN) for 12 h at 4°C on a rotating wheel. After extensive washing, the immunocomplexes were boiled for denaturation and subjected to western blotting.

### Cellular immunofluorescence (IF)

The 4% formalin fixed HEK 293T cells were permeabilized with 0.1% Triton X-100 buffer (Sigma–Aldrich) at room temperature (RT) for 15 min. Cells were blocked with 5% bovine serum albumin (BSA) (Sigma–Aldrich, V900933) in PBS for 60 min at RT and incubated with NFAT1 and FOXC2 monoclonal antibodies overnight at 4°C. After being washed thrice with PBS, respective fluorophore-conjugated secondary antibodies were incubated for 60 min at RT. Cells were then washed and counterstained with DAPI (Beyotime, C1005). The fluorescence was captured under fluorescence confocal microscope (Carl Zeiss, Jena).

### Luciferase reporter gene assay

HEK 293T cells were seeded in 12-well plates and further cultured overnight. The cells were transfected by jetPRIME reagent with luciferase reporter gene plasmids pGL4.27–3xhARRE2s, pGL4.27–3xmut1-hARRE2s, or pGL4.27–3xmut2-hARRE2s, Renilla luciferase plasmid pGL4.74, and ectopic expression plasmids pcDNA3.1–NFAT1, pcDNA3.1–FOXC2, or pcDNA3.1–FOXC2^4A^. After 24 h of transfection, dual Luciferase Reporter Assay kit (Beyotime, RG027) was applied to detect the luciferase activity on a luminometer (Perkin Elmer, EnVision) according to the manufacturer’s protocol. The transcriptional activity was quantified by the ratio of firefly luciferase intensity to Renilla luciferase intensity.

### RNA extraction and real-time PCR

Total RNA was extracted from HEK 293T cells by using TRIzol reagent (Invitrogen, 15596026CN) according to the manufacturer’s instructions. With the use of HiScript IV RT SuperMix Reagent Kit (Vazyme, R423-01), reverse transcription was conducted to generate cDNA. SupRealQ Purple Universal SYBR qPCR Master Mix (Vazyme, Q412-02) and a LightCycler 480 Detection System (Roche) were utilized to carry out real-time PCR analysis. Relative expression of target genes was evaluated by 2-ΔΔCt method. GAPDH served as normalization control. The primer sequences used for real-time PCR are listed in Supplementary Table S2.

### Western blotting analysis

Experiments were conducted using the following primary antibodies: IL2 monoclonal antibody (Proteintech, 60306-1-Ig, 1:1000 dilution), GST-tag antibody (Cell Signaling Technology, 2625S, 1:1000 dilution), His-tag antibody (Abbkine, ABT2050, 1:5000 dilution), Flag-tag antibody (Cell Signaling Technology, 8146 T, 1:1000 dilution), HA-tag antibody (Proteintech, 66 006, 1:5000 dilution), human FOXC2 antibody (RD, MAB5044, 10 μg/ml), and human NFAT1 antibody (Cell Signaling Technology, 5861S, 1:200 dilution). The secondary antibodies HRP-conjugated goat anti-mouse IgG (Abbkine, A21010, 1:5000 dilution), HRP-conjugated goat anti-rabbit IgG (Abbkine, A21020, 1:5000 dilution), goat anti-rabbit IgG-AlexaFluor 488 (Absin, abs20025, 1:200 dilution), and donkey anti-mouse IgG-AlexaFluor 594 (Absin, abs20017, 1:200 dilution) were used. These antibodies were confirmed by western blotting according to the manufacturer’s website. Bands were visualized by enhanced chemiluminescence detection reagents (ECOTOP, EK-5008).

### Statistics and reproducibility

For all immunoblotting experiments, two independent replicates were performed with similar results, and a representative experiment is shown. For assays of ITC, SPR, qRT-PCR, and dual luciferase reporter, all data are expressed as the mean ± SEM or mean ± SD for three independent experiments, and the exact number of replicates (*n*) is shown in the figure legend. Data were analyzed by one-way ANOVA followed by Dunnett’s multiple comparisons test. Statistical analyses were performed using GraphPad Prism 9.0.

## Results

### NFAT1 dominates DNA engagement at composite NFAT–FOX elements

**Figure 1.**
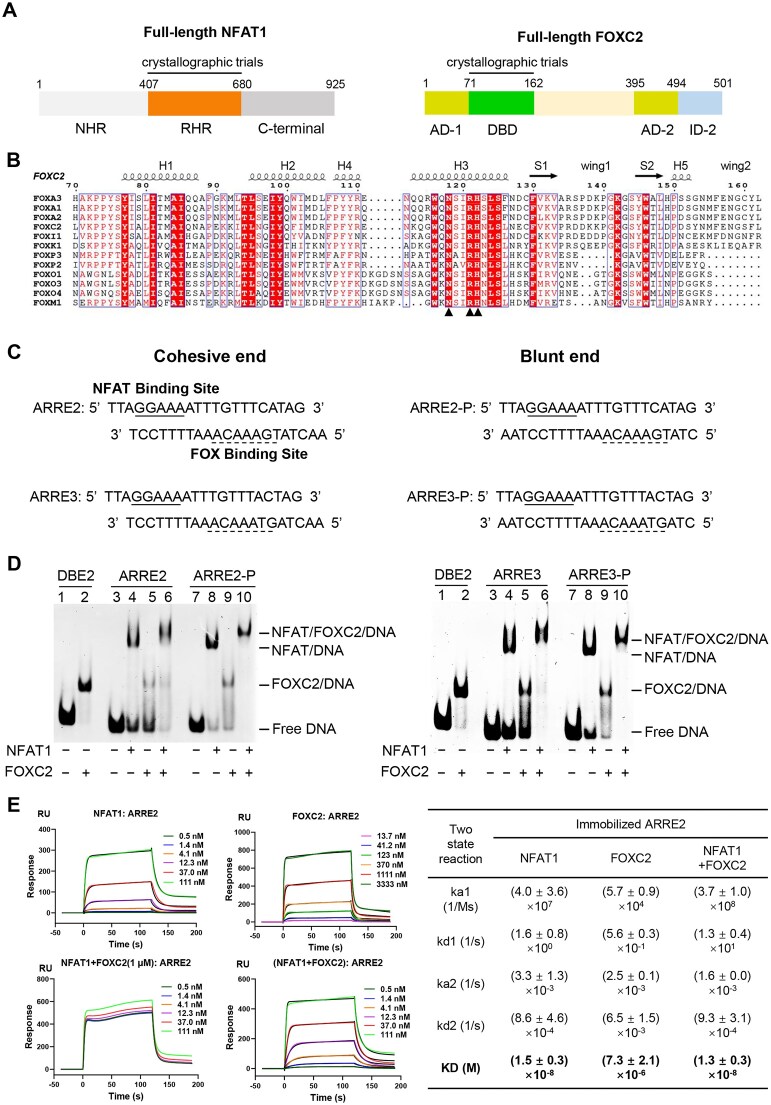
NFAT1 dominates DNA engagement at composite NFAT–FOX elements. (**A**) Structural domains of full-length NFAT1 and FOXC2. (**B**) Alignment of DNA-binding forkhead domains (DBDs) across FOX family proteins. The black triangles indicate the conserved amino acids in H3 responsible for DNA base recognition. (**C**) Sequences of the mouse ARRE2 and its modified variant, with core binding sites underlined. (**D**) EMSA analysis of NFAT1–RHR and FOXC2–DBD binding to ARRE2-related DNAs. DBE2 (Daf 16 family protein-binding element, CAAAATGTAAACAAGA) was used as a negative control. Protein and DNA were combined at ratio of 1:1. (**E**) Quantification of protein–DNA binding affinity by SPR. Representative sensor response curves (colored lines) show binding responses at three-fold serial dilutions of NFAT1 or FOXC2 to immobilized ARRE2 DNA. Black lines represent the global fit to a two-state reaction model. The derived kinetic constants (*k*_a_ and *k*_d_) and equilibrium dissociation constants (*K*_D_) are summarized as mean ± SD in the adjacent table, based on 2–3 independent experiments.

Human NFAT proteins share a conserved N-terminal NFAT-homology region (NHR, residues 1–407 in NFAT1), a Rel homology domain (RHR, residues 408–680 in NFAT1) responsible for DNA binding that is divided into two subdomains, RHR-N and RHR-C, and a C-terminal region [29] (Fig. 1A, left). Human FOXC2 is a multi-domain transcription factor containing an N-terminal transactivation domain 1 (AD-1, residues 1–70), a core DNA-binding domain (DBD, residues 71–162), and a second transactivation domain (AD-2, residues 395–494) linked to an inhibitory region (ID-2, residues 495–501) at the C-terminal region (Fig. 1A, right) [30]. The DBD exhibits high degree of sequence conservation across the FOX family, particularly in the H3 helix, which is essential for DNA recognition (Fig. 1B) [31].

The human and mouse ARRE2 element within *IL2* promoter each contain an identical NFAT-binding motif (GGAAA) adjacent to a FOX consensus sequence (TGAAACA), differing only in the linker region between the two motifs. The sequences shown are derived from the native *IL2* promoter, with minor terminal modifications for experimental purposes (Fig. 1C and Supplementary Fig. S1A) [10]. EMSA experiments were first used to visualize complex formation. Compared to the strong binding of FOXC2 to the canonical FOX-binding element DBE2 (GTAAACA) [24], FOXC2 alone exhibited weaker affinity for both mouse and human ARRE2 element, whereas NFAT1 alone bound robustly (Fig. 1D left, lane 5, and Supplementary Fig. S1B). When co-incubated, NFAT1 and FOXC2 formed a distinct, higher-order ternary complex, accompanied by a reduction in the FOXC2-only signal (Fig. 1D, left, lanes 3–6 and Supplementary Fig. S1). Because the linker length had minimal influence on complex formation, subsequent experiments used the mouse ARRE2 element, consistent with prior studies [14].

To quantify binding and test for cooperativity, we performed SPR assays using an immobilized biotinylated ARRE2. All sensor response curves were best fit by a two-state reaction model (bimolecular association followed by a conformational change). NFAT1 alone exhibited high-affinity, rapid binding (*K*_D_ = 15 nM; ka1 = 4.0 × 10^7^ 1/Ms). FOXC2 alone bound weakly (*K*_D_ = 7.3 µM) with slow kinetics (*k*_a1_ = 5.7 × 10^4^ 1/Ms) (Fig. 1E). When NFAT1 was titrated in the presence of a fixed concentration of FOXC2 (1 μM), the response did not exceed that of NFAT1 alone, and at low NFAT1 concentrations the signal plateaued at the level of FOXC2 background binding. To avoid this complication, we instead titrated pre-formed NFAT1/FOXC2 complexes (1:1 molar ratio). The measured affinity (*K*_D_ = 13 nM) was comparable to that of NFAT1 alone, demonstrating no cooperative enhancement of NFAT1 DNA–binding affinity in the presence of FOXC2.

We next asked if strengthening the FOX motif could induce cooperativity. First, we converted the cohesive ends of ARRE2 to blunt ends (ARRE2-P) (Fig. 1C, top). This modification did not significantly improve FOXC2-only binding (Fig. 1D, left, lanes 7–10, Supplementary Fig. S2A). We then replaced the native FOX consensus motif (TGAAACA) with the canonical sequence (GTAAACA), generating both cohesive-end (ARRE3) and blunt-end (ARRE3-P) variants (Fig. 1C, bottom). As expected, ARRE3 or ARRE3-P significantly improved FOXC2’s intrinsic affinity (*K*_D_ = 2.8 and 1.4 μM, respectively) (Fig. 1D, right, and Supplementary Fig. S2B and C). Notably, although ARRE3/ARRE3-P contains the same core sequence as the DBE2 element, FOXC2 binding to ARRE3/ARRE3-P remained substantially weaker than to DBE2 in EMSA, likely due to differences in flanking sequences. EMSA again showed a single, stable NFAT/FOXC2/DNA ternary complex. However, SPR analysis revealed that the affinity of the NFAT1/FOXC2 complex for these optimized elements was not enhanced, and was slightly reduced compared to NFAT1 alone, providing no evidence of synergistic DNA binding (Supplementary Fig. S2).

Taken together, these data establish that NFAT1 serves as the dominant DNA-binding component at composite NFAT–FOX elements. FOXC2 can be recruited into the complex but does not confer cooperative DNA-binding enhancement, regardless of the intrinsic strength of the FOX site. Thus, NFAT1 dictates the assembly and stability of the NFAT1/FOXC2 complex on DNA.

### Structures of NFAT1–FOXC2–DNA complexes reveal a noncanonical assembly mode

**Figure 2.**
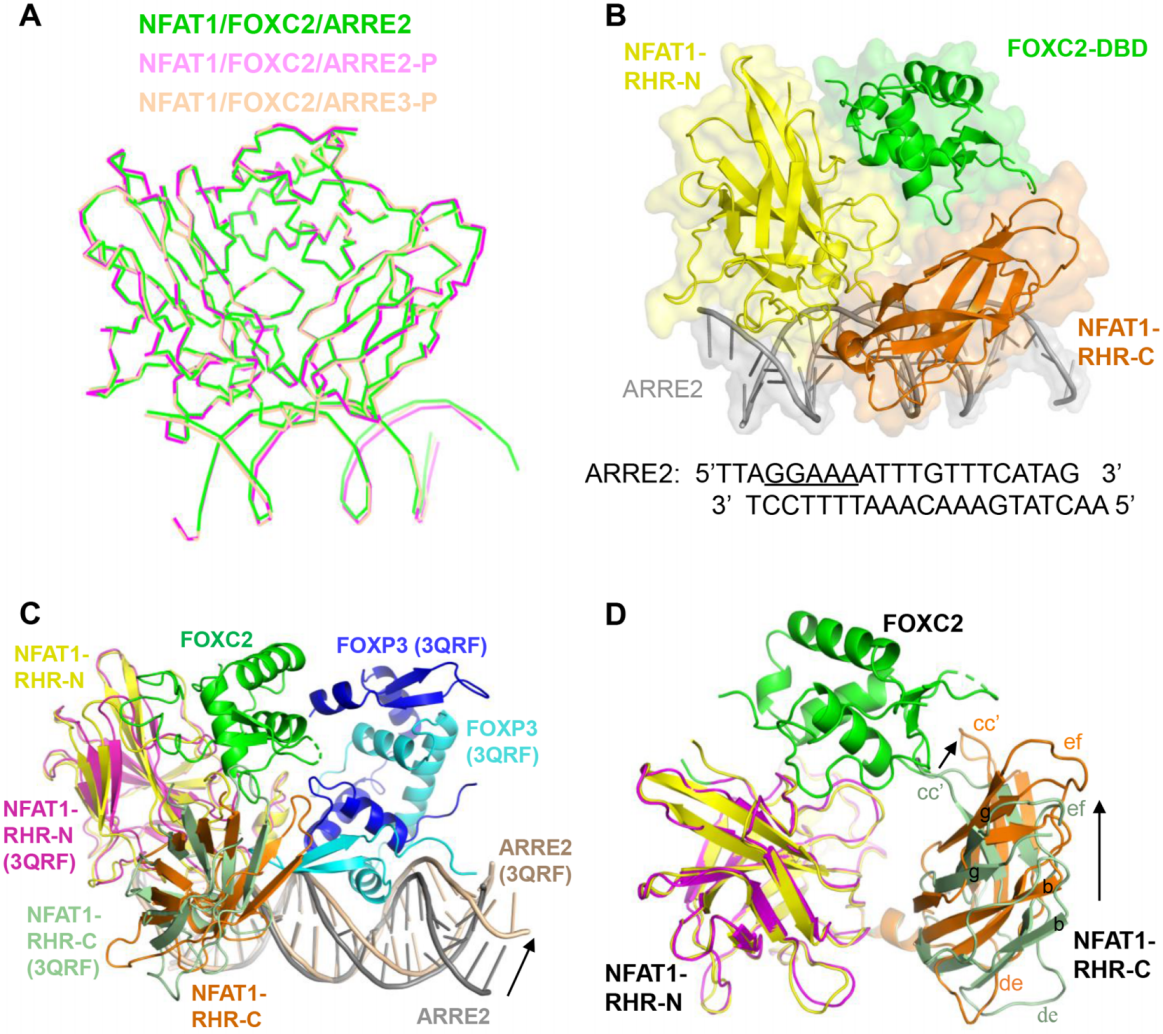
Crystal structures of NFAT1–RHR/FOXC2–DBD/DNA complex. (**A**) Superposition of the three crystal structures of the NFAT1–RHR/FOXC2–DBD/DNA complex. The structures are shown as ribbons. (**B**) Cartoon representation of the NFAT1–RHR/FOXC2–DBD/ARRE2 structure, with FOXC2–DBD, NFAT1–RHR-N, NFAT1–RHR-C, and ARRE2 DNA. (**C**) Structural alignment comparing this study NFAT1–RHR/FOXC2/ARRE2 complex with the previously reported NFAT1–RHR/FOXP3 dimer/ARRE2 complex (PDB: 3QRF). (**D**) Conformational comparison of NFAT1-RHR between the FOXC2-bound (this study) and FOXP3-bound (3QRF) complexes.

To gain structural insight into how NFAT1 and FOXC2 assemble on DNA in the absence of binding cooperativity, we determined crystal structures of NFAT1–RHR/FOXC2–DBD complexes with three distinct ARRE elements (ARRE2, ARRE2-P, and ARRE3-P). All structures were solved at 2.6–2.8 Å resolution in the P 2_1_2_1_2_1_ space group, with one NFAT1–RHR/FOXC2–DBD/DNA ternary complex in the asymmetric unit (Supplementary Table S1). Surprisingly, despite differences in DNA terminal structures (cohesive versus blunt ends) and FOX binding motifs (consensus TGAAACA versus canonical GTAAACA), these complexes exhibited nearly identical architectures with an overall RMSD of 0.5–0.6 Å (Fig. 2A). Remarkably, the ternary complex persisted even upon disruption of the FOX recognition motif in ARRE2 (Supplementary Fig. S3), demonstrating that FOXC2 binding occurs independently of its cognate DNA sequence.

Structural analysis of the NFAT1–RHR/FOXC2–DBD/ARRE2 complex revealed an unanticipated binding mode. While NFAT1–RHR formed a characteristic V-shaped groove to engage the GGAAA motif, FOXC2–DBD unexpectedly bypassed its putative binding site and instead inserted into the NFAT1 groove (Fig. 2B and Supplementary Fig. S4). This interaction occurred while FOXC2–DBD maintained an overall fold similar to its DNA-bound conformation (RMSD 0.5 Å versus PDB: 6AKO), albeit with significant rearrangements in the wing 1 and wing 2 regions (Supplementary Fig. S5).

Comparative structural analysis highlights striking differences between FOXC2 and other FOX family members. In contrast to FOXP proteins that bind DNA as monomers/dimers and contact NFAT1 through wing 1 and H2 helix (inducing DNA bending), FOXC2 interacts directly with NFAT1 without requiring the FOX motif (Fig. 2C and Supplementary Fig. S6). Notably, NFAT1–RHR-C exhibited remarkable conformational plasticity to accommodate FOXC2 binding. Structural comparisons reveal outward flipping of the cc' loop, along with significant displacements of the ef loop, de loop, and β-sheets b/g, resulting in an expanded V-shaped groove (Fig. 2D). These observations underscore NFAT1’s structural adaptability in forming distinct transcriptional complexes with different partner proteins.

Together, our structural studies reveal a noncanonical assembly mode in which FOXC2 interacts with NFAT1 predominantly through protein–protein interactions, rather than through cooperative DNA binding. This architecture provides a structural basis for the dominant role of NFAT1 in the assembly and stabilization of the NFAT1/FOXC2 complex on composite DNA elements.

### NFAT1 recruits FOXC2 through direct protein–protein interaction

**Figure 3.**
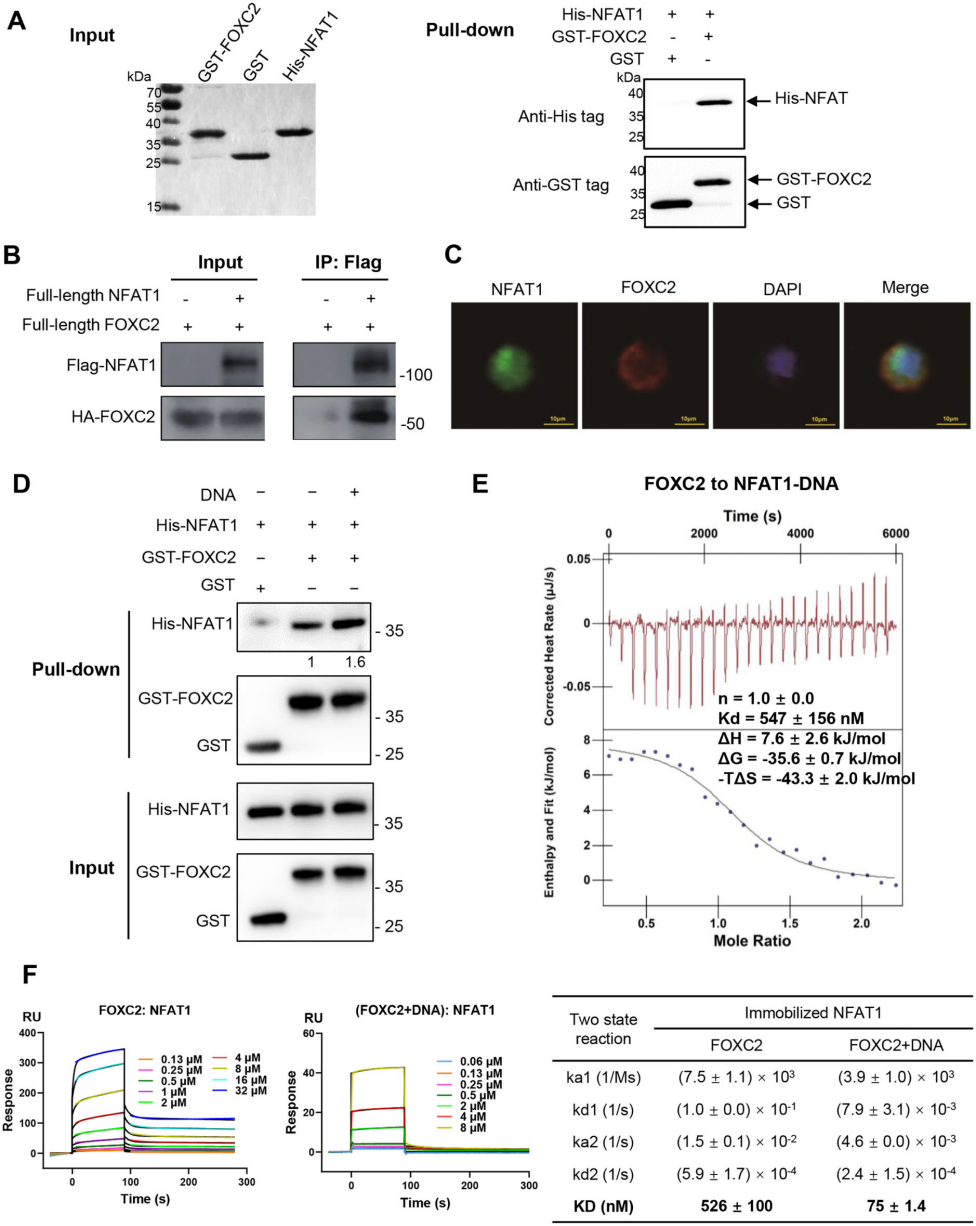
NFAT1 recruits FOXC2 through direct protein–protein interaction, enhanced by DNA binding. (**A**) GST pull-down assay demonstrating FOXC2–NFAT1 interaction. GST or GST–FOXC2–DBD was incubated with His–NFAT1–RHR, followed by SDS–PAGE (input, left) and western blotting (WB) of pull-down complexes using anti-GST and anti-His antibodies. The results of two independent replicates were similar, with one representative experiment being shown. (**B**) Co-IP of full-length NFAT1 and FOXC2. HEK 293T cells were co-transfected with Flag–NFAT1 and HA–FOXC2, followed by immunoprecipitation (IP) with Flag beads and western blot analysis. (**C**) Representative fluorescent photographs of immunofluorescence staining for NFAT1 (green) and FOXC2 (red) in HEK 293T cells. Scale bar: 10 μm. (**D**) GST pull-down assay with NFAT1, FOXC2, and DNA (1:1:1 molar ratio). Two independent replicate experiments were performed, and the figure shows a representative experiment. Semi-quantitative band densitometry was performed, and the relative binding, normalized to the no-DNA control, was shown below. (**E**) Representative ITC heat signals and the integrated curves for FOXC2 binding to a preformed NFAT1–DNA complex (1:1 molar ratio). The integrated curve subtracted the background heat signals of FOXC2 titration buffer. Data represent mean ± SD from three independent experiments. (**F**) Quantitative SPR analysis of NFAT1 binding to FOXC2 or to a pre-formed FOXC2–DNA complex (DNA fixed at 16 μM). Sensor response curves (colored curves) were globally fitted to a two-state reaction model (black curves). The derived kinetic and equilibrium constants (mean ± SD, from two independent experiments) are summarized in the adjacent table.

The structural interface observed between NFAT1–RHR and FOXC2–DBD in our crystallographic studies led us to systematically examine the DNA-dependence of this interaction. Using GST pull-down assays, we demonstrated that GST–FOXC2–DBD specifically interacts with NFAT1–RHR in the complete absence of DNA, while control GST showed no detectable binding (Fig. 3A). This *in vitro* evidence of DNA-independent complex formation directly validates the protein–protein interaction interface observed in our ternary crystal structures.

To extend these findings to a cellular context, we performed co-immunoprecipitation experiments in HEK 293T cells. Full-length HA-tagged FOXC2 robustly co-precipitated with Flag-tagged NFAT1, confirming that the interaction occurs between properly folded, post-translationally modified proteins in a physiological environment (Fig. 3B). Live-cell imaging further revealed prominent co-localization of fluorescently labeled NFAT1 (green) and FOXC2 (red) at the nuclear periphery (Fig. 3C), a pattern consistent with their potential engagement in transcriptionally active nuclear regions. Together, these integrated structural, biochemical, and cellular data establish that NFAT1 recruits FOXC2 primarily through direct protein–protein interaction, independent of DNA tethering. This recruitment mechanism is consistent with our quantitative binding analyses, which show that FOXC2 does not enhance NFAT1’s DNA-binding affinity, but rather associates with DNA-bound NFAT1 to form a stable ternary complex.

### DNA-mediated conformational stabilization of NFAT1 facilitates FOXC2 recruitment

Structural analysis revealed that NFAT1–RHR adopted characteristic V-shaped groove conformation when bound to the ARRE2 element, formed through cooperative interactions between the RHR-N and RHR-C domains [6, 10] (Fig. 2). While RHR-N has been shown to mediate specific DNA recognition (a conserved feature across NFAT1/DNA complexes) [7, 10, 14, 32], we have observed that RHR-C exhibits significant conformational flexibility (Fig. 2D and Supplementary Fig. S7). This structural plasticity suggested that DNA binding might stabilize an NFAT1 conformation more favorable for FOXC2 recruitment.

To test this hypothesis, we performed comparative binding analyses. GST pull-down assays first confirmed that NFAT1 and FOXC2 interact in a DNA-independent manner (Fig. 3D). To quantitatively assess whether DNA enhances this interaction, we employed isothermal titration calorimetry (ITC). Three independent ITC experiments consistently showed that FOXC2 bound free NFAT1 with moderate affinity (*K*_d_ = 3.0 ± 0.3 μM) in an endothermic reaction (Δ*H* > 0), whereas its binding to the pre-formed NFAT1–ARRE2 complex was substantially stronger (*K*_d_ = 547 ± 156 nM), representing an approximately 5.5-fold increase in affinity (Fig. 3E, and Supplementary Figs S8 and 9).

To obtain kinetic insight and validate these observations with an orthogonal method, we performed SPR assays. NFAT1 was immobilized on the sensor chip, and FOXC2 binding was measured in the absence or presence of ARRE2 DNA. The Sensor response curves were again best fit by a two-state reaction model (Fig. 3F). The presence of DNA markedly enhanced the interaction, reducing the overall KD from 526 nM (FOXC2 alone) to 75 nM (FOXC2 with DNA), showing an approximately 7-fold increase. Kinetic decomposition indicated that this enhancement primarily resulted from stabilization of the initial bimolecular complex (*k*_d1_ decreased from 1.0 × 10^−1^ 1/s to 7.9 × 10^−3^ 1/s). These SPR measurements, together with the ITC results, provide robust and reproducible evidence that DNA binding to NFAT1 stabilizes a conformational state that presents a more favorable interface for FOXC2 recruitment. Therefore, our integrated data demonstrate that ARRE2 DNA not merely serves as the primary binding site for NFAT1 but also conformationally stabilizes NFAT1 in a manner that significantly enhances FOXC2 binding and ternary-complex stability.

### Two regions within a single interface mediate NFAT1–FOXC2 complex formation

**Figure 4.**
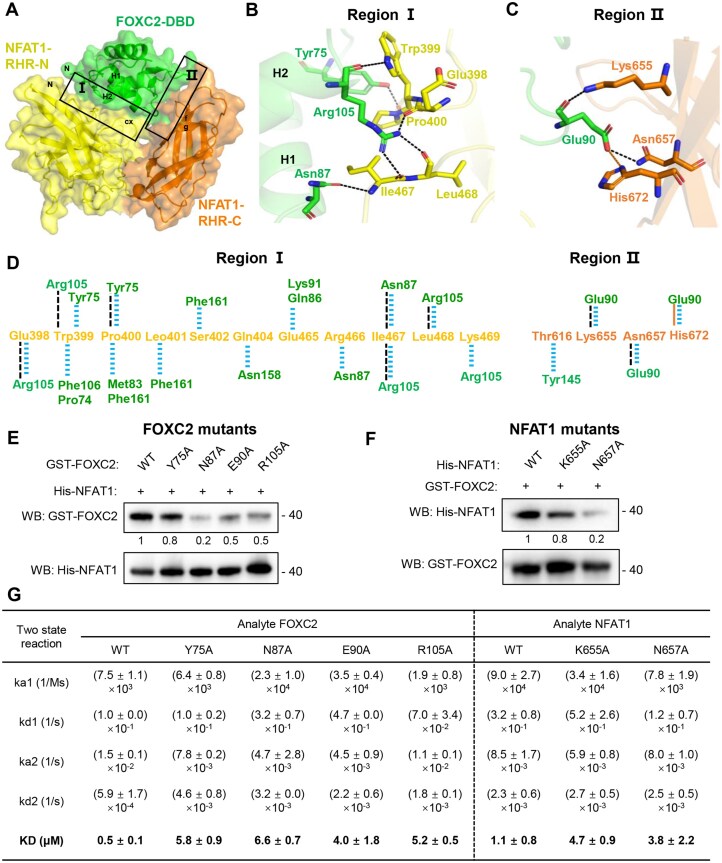
Two regions within a single interface mediate NFAT1–FOXC2 complex formation. (**A**) Structural overview of FOXC2–DBD and NFAT1–RHR interaction regions. (**B** and **C**) Detail interactions of region Ⅰ (B) and region Ⅱ (C), with key residues shown as sticks and hydrogen bonds indicated by black dashed lines. (**D**) Schematic representation of FOXC2–DBD/NFAT1–RHR interactions, with hydrogen bonds shown as black dashed lines (<3.5 Å), salt bridges as orange solid lines (<3.5 Å), and other non-bonded contacts shown as blue dashed lines (<4.0 Å). (**E** and **F**) Pull-down assays validating the regions: (E) Wild-type or mutant GST–FOXC2 incubated with His–NFAT1 (captured by Ni-NTA beads), and (**F**) wild-type or mutant His–NFAT1 incubated with GST–FOXC2 (captured by Glutathione–Sepharose), followed by western blot analysis. A representative result from two independent replicates is shown, with semi-quantitative binding relative to wild-type (WT) indicated below. (**G**) Quantitative binding analysis of WT and mutant protein interactions using SPR experiments. The kinetic and equilibrium dissociation constants are summarized in the table. Data are presented as mean ± SD from two independent experiments.

To show the detailed interactions of NFAT1 and FOXC2, we then analyzed the NFAT–RHR V-shaped groove and FOXC2–DBD interfaces. NFAT1–RHR engaged FOXC2–DBD through two regions within a single continuous interface (Fig. 4A). Region Ⅰ involved the N-terminal region and cx loop of RHR-N interacting with the N-terminus and the junction of helices H2 and H4 of FOXC2–DBD, while region II comprised the f and g β-sheets of RHR-C contacting the H1–H2 connecting loop of FOXC2–DBD. Detailed examination showed that in region I, FOXC2 Arg105 formed multiple hydrogen bonds and extensive van der Waals contacts with RHR-N residues Glu398, Trp399, Ile467, and Leu468 (Fig. 4B and D, left). Additional stabilization came from a hydrogen bond between FOXC2 Tyr75 and RHR-N Pro400, as well as an interaction between FOXC2 Asn87 and the backbone of RHR-N Leu468. Region II featured particularly strong interactions, with FOXC2 Glu90 forming both hydrogen bonds and a salt bridge with RHR-C residues Lys655, Asn657, and His672 (Fig. 4C and D, right). These specific contacts were complemented by numerous van der Waals interactions that collectively stabilized the NFAT1–FOXC2 complex.

To validate these structural observations, we performed structure-based mutational analysis targeting key interfacial residues. Pull-down assays with FOXC2 mutants (Y75A, N87A, E90A, and R105A) showed significantly reduced binding to NFAT1–RHR compared to wild-type counterpart (Fig. 4E). Similarly, NFAT1–RHR mutants (K655A and N657A) exhibited impaired interaction with FOXC2 (Fig. 4F). We further quantified the impact of these mutations using SPR. When NFAT1 was immobilized, the binding affinity of wild-type FOXC2 was 0.5 µM (Fig. 3F). In contrast, the FOXC2 interfacial mutants displayed substantially weakened affinities, ranging from 4.0 to 6.6 µM, corresponding to an 8- to 13-fold reduction (Fig. 4G, left, and Supplementary Fig. S10A). Conversely, when FOXC2 was immobilized, wild-type NFAT1 bound with an affinity of 1.1 µM. The NFAT1 mutants K655A and N657A showed 3- to 4-fold reductions in affinity, with *K*_D_ values of 4.7 and 3.8 µM, respectively (Fig. 4G, right, and Supplementary Fig. S10B). These biochemical results precisely corroborate our structural findings, confirming that both regions are essential for stable complex formation. Together, these findings establish that NFAT1 recruits FOXC2 through a dual-region mechanism, where each contact surface contributes distinct but complementary interactions to ensure robust complex assembly.

### FOXC2 acts as a co-repressor to repress the transcription of NFAT1-driven genes

**Figure 5.**
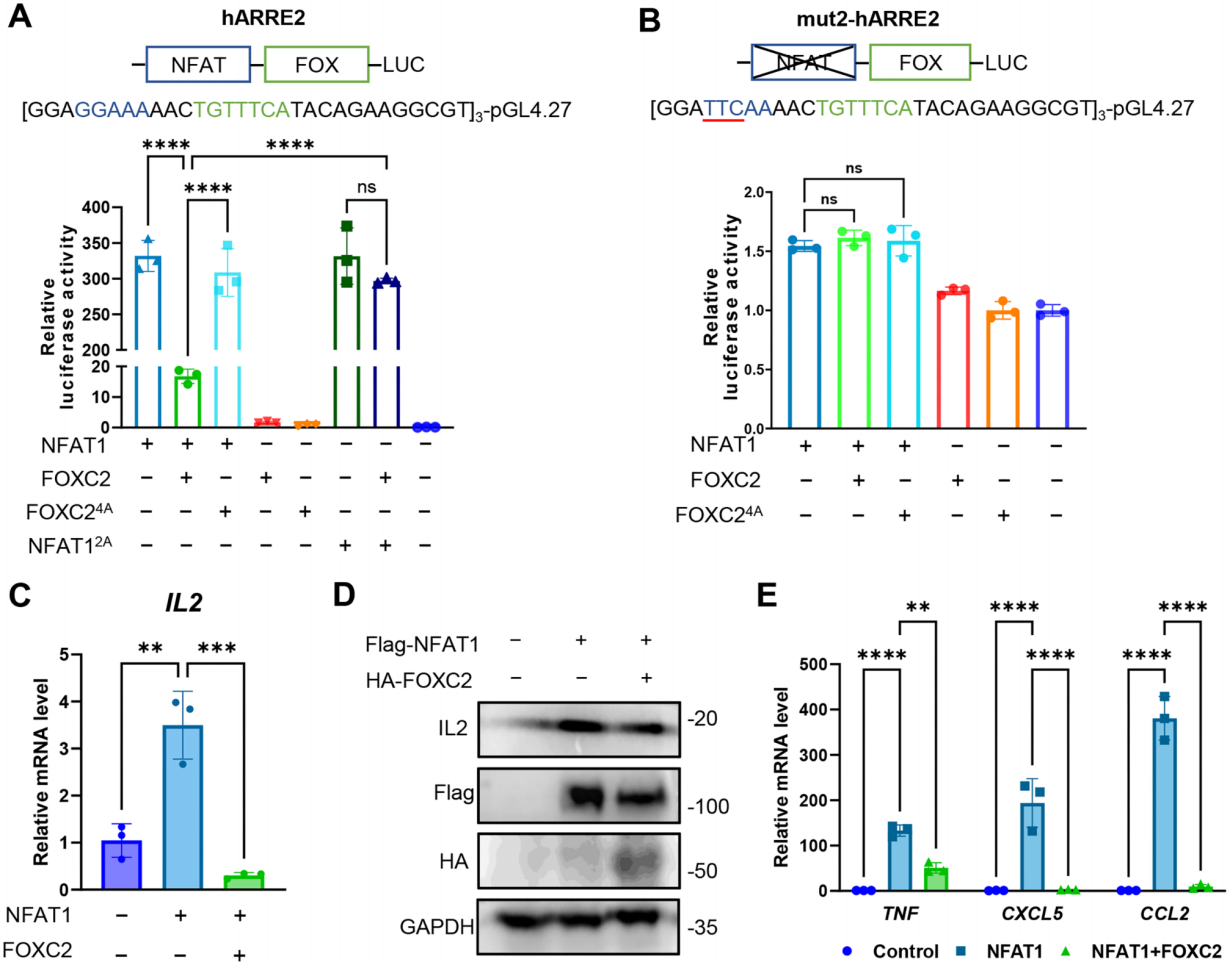
FOXC2 acts as a co-repressor to repress the transcription of NFAT1-driven genes. (**A**) Dual luciferase reporter assay demonstrating that disruption of the FOXC2–NFAT1 interaction interface impairs FOXC2-mediated transcriptional repression of NFAT1 on the *IL2* promoter (ARRE2 element) in HEK 293T cells. The sequences of the reporter constructs are displayed at the top of the figures. Renilla values were used as reference. Values were normalized to empty plasmid group. (**B**) Mutation of the intrinsic NFAT binding site in hARRE2 (mut2-hARRE2) abolishes both NFAT1-driven transcriptional activation and FOXC2-mediated repression. (**C**) qRT-PCR analysis of *IL2* mRNA levels in HEK 293T cells overexpressing NFAT1 and/or FOXC2, normalized to GAPDH (internal control for all qRT-PCR assays). (**D**) Corresponding reduction in IL2 protein levels upon FOXC2 co-expression, as determined by western blot. (**E**) qRT-PCR profiling of additional NFAT1-regulated genes exhibiting FOXC2-mediated transcriptional repression. All of data are shown the mean ± SD of *n* = 3 independent replicates. *P*-values were calculated using the transfected empty plasmid group as a control. ** *P* < 0.01, *** *P* < 0.001, **** *P* < 0.0001, ns: not statistically significant (*P* > 0.05).

To establish the functional effects of NFAT1–FOXC2 interaction, we performed comprehensive transcriptional analyses using luciferase reporter assays and endogenous gene expression profiling. Reporter assays in HEK 293T cells demonstrated that NFAT1 alone strongly activated transcription from the *IL2* promoter ARRE2 element (consistent with our EMSA and SPR results), while co-expression with wild-type FOXC2 caused a dramatic 20-fold repression of NFAT1-mediated activation (Fig. 5A). Importantly, the FOXC2 interface 4A mutant (Y75A/N87A/E90A/R105A) and the corresponding NFAT1 interface 2A mutant (K655A/N657A) significantly attenuated this repressive effect, directly linking the structural interaction interface to functional outcomes. The repressive activity of FOXC2 showed a clear concentration dependent pattern (Supplementary Fig. S11) and required intact NFAT1 binding sites, as mutation of the NFAT recognition motif in ARRE2 abolished both activation by NFAT1 alone and repression by the NFAT1–FOXC2 complex (Fig. 5B and Supplementary Fig. S12). Notably, mutation of the FOX-binding site did not impair FOXC2-mediated repression (Supplementary Fig. S13), consistent with our structural and biochemical evidence that FOXC2 recruitment is independent of DNA binding.

Parallel qRT-PCR analysis of endogenous *IL2* expression confirmed these findings, with NFAT1 overexpression increasing *IL2* mRNA levels approximately 3.5-fold, while NFAT1–FOXC2 co-expression reduced expression below baseline levels (Fig. 5C). Western blot analysis similarly showed that FOXC2 co-expression counteracted NFAT1-induced IL2 protein production (Fig. 5D). The repressive effect extended to multiple NFAT1 target genes, including *TNF, CXCL5*, and *CCL2*, with particularly strong suppression of chemokine *CXCL5* and *CCL2* expression (Fig. 5E). These results establish FOXC2 as a broad-spectrum co-repressor of NFAT1-driven transcription, capable of modulating diverse immunological gene expressions through its direct interaction with NFAT1. The DNA-independent nature of this repression mechanism suggests FOXC2 may serve as a versatile negative regulator of NFAT1 activity across different genomic contexts.

### Transcriptional suppression of NFAT1 through a broader FOX–NFAT1 interaction

**Figure 6.**
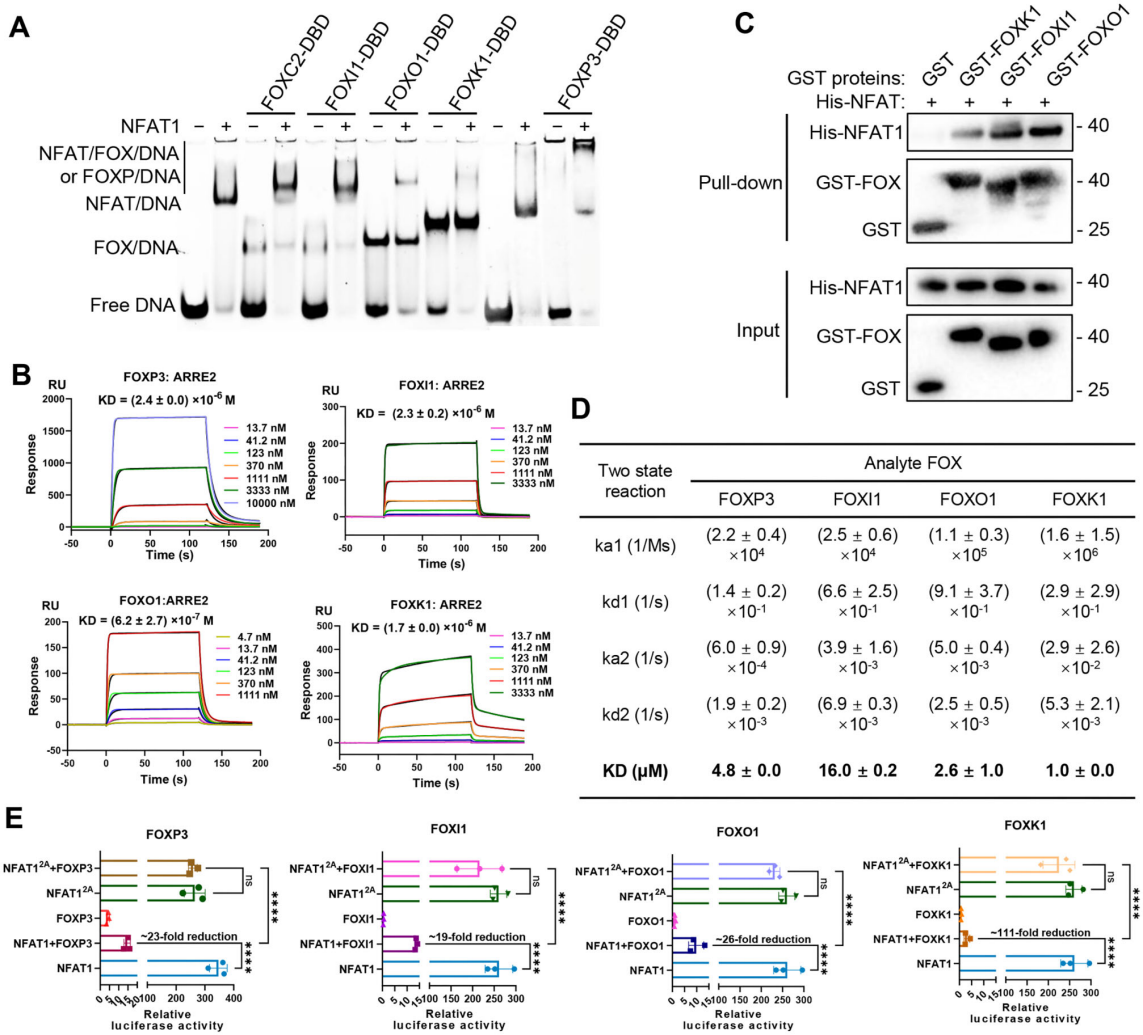
Broad FOX family-mediated suppression of NFAT1 transcriptional activity. (**A**) EMSA analysis of FOX family members binding to NFAT1 and ARRE2 DNA. (**B**) SPR analysis of the binding affinities of FOX to immobilized ARRE2 DNA. Representative sensor response curves (colored curves) show binding responses, which were globally fitted to a two-state reaction model (black curves). Data are from two independent experiments. (**C**) GST pull-down assay demonstrating interactions between His–NFAT1 and GST-tagged FOXK1, FOXI1, or FOXO1. Complexes were captured using Glutathione–Sepharose and detected by immunoblotting. A representative result from two independent replicates is shown. (**D**) Summary of the kinetic and equilibrium constants for the interactions between various FOX proteins and NFAT1, as determined by SPR. Data are presented as mean ± SD from two independent experiments. (**E**) Dual luciferase reporter assay assessing the repressive effects of various FOX proteins on NFAT1-driven *IL2* promoter (ARRE2) activity in HEK 293T cells. All of data are shown the mean ± SD of *n* = 3 independent replicates. *P*-values were calculated using the empty plasmid group as a control. **** *P* < 0.0001.

Building upon our characterization of FOXC2 as an NFAT1 co-repressor, we sought to determine whether this regulatory mechanism extends more broadly across the FOX transcription factor family. We first conducted a qualitative assessment of complex formation via EMSA. The EMSA assays demonstrated that multiple FOX proteins (including FOXO1, FOXI1, FOXK1, and FOXP3) could form NFAT1-containing complexes on ARRE2, with markedly different apparent stabilities (Fig. 6A). Among these, FOXP3 exhibited a distinct propensity to form oligomeric complexes, consistent with prior reports of NFAT–FOXP cooperative DNA binding [10, 14, 33]. To quantitatively assess DNA binding of these FOX proteins, we performed SPR analysis. All tested FOX bound ARRE2 with low, micromolar-range affinities (Fig. 6B), mirroring the pattern observed for FOXC2. Furthermore, direct protein–protein interactions between NFAT1 and these FOX family members were validated by GST pull-down (Fig. 6C) and quantified by SPR, revealing a spectrum of binding strengths (Fig. 6D).

To assess functional outcomes, we performed dual luciferase reporter gene assays. All tested FOX proteins significantly suppressed NFAT1-driven from the *IL2* promoter, with FOXK1 showed the strongest repression (∼111-fold) and FOXP3 also showing potent inhibition (∼23-fold), in line with its known role in Treg suppression (Fig. 6E). Strikingly, FOXI1, FOXO1, FOXK1, and FOXP3 all failed to repress transcription when co-expressed with an NFAT1^2A^ mutant (K655A/N657A) that disrupts the FOXC2-binding interface (Fig. 6E), suggesting that these diverse FOX proteins engage NFAT1 through a common or highly overlapping surface on NFAT1. Control experiments established that this repressive activity strictly required an intact NFAT binding site but remained unaffected by mutations to the FOX recognition element (Supplementary Fig. S14), consistent with our structural model of DNA-independent FOX–NFAT1 complex formation.

Together, this functional analysis revealed the broader FOX–NFAT1 interaction network possesses consistent transcriptional regulatory properties, wherein multiple FOX family members can engage NFAT1 through conserved protein–protein patterns to modulate its transcriptional output. The varying degrees of repression observed among different FOX proteins (FOXK1 > FOXO1 > FOXP3 > FOXC2 > FOXI1) suggest this network may enable nuanced, context-dependent control of NFAT1 activity across different cellular environments.

## Discussion

Our study identifies FOXC2 as a transcriptional co-repressor of NFAT1, uncovering a previously unrecognized mechanism in which FOXC2 engages DNA-bound NFAT1 through a DNA-facilitated protein–protein interface. Unlike previously characterized FOXP–NFAT1 assemblies that rely on composite DNA motifs, FOXC2 lacks direct DNA recognition and instead docks onto a V-shaped groove of the NFAT1–RHR domain that forms in the DNA-bound state (Fig. 2). This distinct structural arrangement highlights how the local DNA context, such as the three-base-pair spacer between NFAT and FOX sites in the ARRE2 motif, can sterically exclude FOXC2 from direct DNA recognition due to its longer wing1 region compared with FOXP (Supplementary Fig. S15). Despite these mechanistic differences, biochemical and function assays show that FOXC2 and FOXP3 share a conserved NFAT1-recruitment logic: both interact with NFAT1 with micromolar affinity in a DNA-independent manner and repress NFAT1-driven transcription (Fig. 6). This indicates that an NFAT1-centric repression module is utilized across FOX subfamilies.

The divergence between FOXC2 and FOXP lies in their assembly mechanisms, oligomeric plasticity, and consequent regulatory selectivity. FOXP3 exhibits pronounced conformational flexibility, with its DNA-binding domain capable of adopting domain-swapped dimer or classical monomeric folds depending on structural context and the presence of flanking regions such as the Runx1-binding region (RBR) [16, 33, 34]. This plasticity enables FOXP3 to recognize diverse DNA motifs through distinct oligomeric states: head-to-head dimers on canonical FOX sites and ladder-like multimers on T_3_G-repeat microsatellites. Mutations that disrupt domain swapping (e.g. W348Q/M370T/A372P) or certain IPEX-associated variants (e.g. R337Q) shift FOXP3’s oligomeric equilibrium, impairing its ability to organize higher-order DNA architectures and diminishing its repression of NFAT-driven targets such as *IL2* [14, 33]. Notably, while the FOXP3–NFAT1 crystal structure did not capture direct contact with NFAT1 residues K655/N657, our functional data show that these residues are essential for FOXP3-mediated repression (Fig. 6E). This suggests that FOXP3 can engage NFAT1 through a broader, functionally conserved interface beyond the crystallographic interaction surface, consistent with its known conformational plasticity. In contrast, FOXP2 primarily adopts a canonical forkhead conformation when interacting with NFAT, as seen in earlier structural studies [10]. Importantly, FOXP3 can reprogram NFAT activity toward immunosuppressive transcriptional programs—a function not robustly observed with FOXP2 [35]. These subfamily–specific structural and functional distinctions highlight how FOXP proteins exploit direct DNA engagement to achieve context–dependent regulatory outcomes.

In contrast, FOXC2 operates through a DNA–induced co–repressor mechanism. Its binding follows a sequential assembly pathway governed by affinity: NFAT1 binds DNA first, and the DNA–stabilized conformation of NFAT1 then recruits FOXC2 to form the ternary complex. This interaction induces an expansion of the NFAT1 V-shaped groove, with the NFAT1–RHR-C domain undergoing significant conformational changes. Notably, this DNA-induced conformational change enhances FOXC2–NFAT1 binding affinity (Fig. 3D–F, and Supplementary Figs S8 and 9). To our knowledge, this is the first reported case of DNA-facilitated enhancement of a protein–protein interaction through structural stabilization rather than shared DNA sequence recognition.

Significantly, the FOXC-like recruitment mode appears to represent a broader functional strategy. Our findings that other FOX family members (FOXI1, FOXO1, and FOXK1) also suppress NFAT1 activity, and that this repression depends on the same conserved NFAT1 interface defined by the FOXC2 complex (Fig. 6E), strongly suggest a shared mechanism of DNA-facilitated co-repression. This extends the biological relevance of our structural model and indicates that the ability to function as an NFAT co-repressor may be a more widespread feature among FOX proteins, particularly those that operate primarily through protein-protein interactions rather than strict cooperative DNA binding.

Together, these findings support a two–route assembly model for NFAT–FOX complexes. In the FOXC–like mode, NFAT serves as the dominant DNA–anchoring factor, and the FOX protein is recruited primarily through protein–protein interaction with DNA–bound NFAT. In the FOXP–like mode, FOX participation in direct DNA engagement is more prominent, enabling cooperative binding on permissive composite motifs (e.g. ARRE2–like spacers). This model reconciles our FOXC2 structure with prior NFAT–FOXP architectures and explains differential regulatory selectivity: FOXP–mediated repression is more constrained by local motif architecture, whereas FOXC–like repression may extend across a broader set of NFAT–occupied elements because it does not require strict composite DNA geometry for FOX–DNA contact. Importantly, the model accommodates the conformational plasticity of FOXP proteins, which can adopt alternative states (e.g. the FOXP2 monomer–like NFAT–associated mode) that still allow DNA engagement while interacting with NFAT.

The FOXC2–NFAT1 interface is extensive and comprises two interaction surfaces mediated by numerous electrostatic and hydrophobic contacts (involving FOXC2 residues Y75, N87, E90, R105, and NFAT1 residues E398, W399, K655, and N657), which are essential for complex formation and transcriptional repression (Figs 4 and 5A). Historically, FOX proteins have been viewed primarily as transcription factors that bind DNA via the H3 helix of their conserved DBD [23, 36–38]. This work provides the first evidence that a FOX protein can act as a transcriptional co-repressor for NFAT1 without recognizing a DNA motif. This mode of regulation contrasts with DNA-dependent FOX dimerization on composite elements (e.g. FOXA1/DIV) and heterodimeric FOX–nuclear receptor assemblies, where FOX factors directly engage DNA [39, 40]. These opposing paradigms underscore the functional versatility of the FOX family, with some members pioneering chromatin through direct DNA contacts while others, like FOXC2, modulate transcription via protein–protein interactions. Thus, FOX proteins can employ distinct strategies for transcriptional regulation, ranging from DNA-dependent pioneers to co-repressors that operate through protein–protein interactions independent of DNA engagement. Although a substantial number of FOX–transcription factor complex motifs have been identified in the human gene regulatory code [41], whether FOX proteins can serve as co-repressors for other classes of transcription factors, as seen with FOXC2–NFAT1, remains an important open question.

Our functional assays further show that repression of NFAT1 by FOXC2 extends to multiple target genes, including IL2, TNF, CXCL5, and CCL2 (Fig. 5C and D). Previous large-scale mass spectrometry analyses have identified FOXK1 and FOXK2 as NFAT-interaction factors, and FOXD3 has been shown to interact with NFAT4 and inhibit NFAT4-driven transcription programs [17, 42]. A concrete next step will be to compare, in parallel, the structural dynamics and genomic occupancy of these FOX proteins when bound to NFAT1, to determine whether variations in interface conformation correlate with distinct gene regulatory programs. Such comparative analyses would clarify both the conserved and divergent aspects of FOX-mediated NFAT repression.

The functional outcome of NFAT signaling is determined by its transcriptional partners. While co-activators such as AP1, GATA3, and MEF2 cooperate with NFAT to activate specific gene expression programs [5, 43, 44], FOX proteins appear to antagonize this activation. In particular, AP, primarily a FOS/JUN heterodimer, forms a tetrameric complex with NFAT1 on composite DNA elements to drive expression of cytokines, chemokines, and metabolic genes during T-cell activation [5, 6]. Intriguingly, FOX proteins and AP1 compete for overlapping binding sites on the IL2 promoter element ARRE2 or NFAT1 (Supplementary Fig. S16). This indicates a competitive model: FOX proteins interfere with the transcriptional output of NFAT1 by blocking AP-1 binding. Supporting this model, disruption of the FOX-binding interface on NFAT1 significantly reduces its transcriptional activity (Supplementary Fig. S13), likely by impairing AP1-mediated co-activation. It should be noted that other important partners including p300 and Runx proteins also contribute to this regulatory network by modulating NFAT1 transcriptional activity, suggesting that the functional outcome of NFAT signaling is determined by a complex interplay between these cofactors [45]. Further studies are needed to elucidate the precise competitive dynamics between AP1 and different FOX partners, as well as their integrated functions with other cofactors such as p300 and Runx in different cellular contexts.

From a broader perspective, this noncanonical co-repressor role of FOXC2 adds a new dimension to FOX biology, which has traditionally regarded FOX proteins solely as transcription factors. Selective repression of NFAT1 without displacing it from DNA offers a potential regulatory strategy in contexts such as immune tolerance, T cell exhaustion, and chronic inflammation, where calibrated transcriptional control is advantageous [4]. The disruption of the interaction of NFAT1 and FOXP3, facilitated by a FOXP3-derived peptide, has been shown to inhibit the conversion of T cell into iTregs and to promote T-cell proliferation, thereby exerting antitumor effects [46]. Therefore, our structural definition of the FOXC2–NFAT1 interface opens opportunities for therapeutic targeting: small molecules or peptides that disrupt this interaction—without affecting NFAT–DNA binding—could achieve tissue- or context-specific immune modulation with fewer side effects than global NFAT inhibition.

In summary, our findings establish DNA-facilitated co-repression as a previously unrecognized mechanism of transcriptional control, redefining the functional scope of FOX proteins, and provide a structural framework in which NFAT–FOX association is context– and subfamily–dependent. This framework reconciles distinct assembly topologies with regulatory output and offers a testable basis for understanding how different FOX factors modulate NFAT activity in health and disease.

## Supplementary Material

gkag367_Supplemental_File

## Data Availability

The coordinates and map files of the NFAT1/FOXC2/DNA complex structures generated in this study have been deposited in the Protein Data Bank database under the accession codes 9VRQ (https://doi.org/10.2210/pdb9vrq/pdb), 9VRT (https://doi.org/10.2210/pdb9vrt/pdb) and 9VS2 (https://doi.org/10.2210/pdb9vs2/pdb). Structure coordinates used in this study are accessible in PDB under the following accession codes: 3QRF (structure of NFAT1/FOXP3/ARRE2 complex, https://doi.org/10.2210/pdb3qrf/pdb), 2AS5 (structure of NFAT1/FOXP2/ARRE2 complex, https://doi.org/10.2210/pdb2as5/pdb), 6AKO (structure of FOXC2/DBE2 complex, https://doi.org/10.2210/pdb6ako/pdb), 1OWR (structure of NFAT1/DNA2 complex, https://doi.org/10.2210/pdb1owr/pdb), 1P7H (structure of NFAT1/DNA3 complex, https://doi.org/10.2210/pdb1p7h/pdb), 1PZU (structure of NFAT1/DNA4 complex, https://doi.org/10.2210/pdb1pzu/pdb), 1A02 (structure of NFAT1/AP1/DNA complex, https://doi.org/10.2210/pdb1a02/pdb), and 7TDX (FOXP3ΔN/DNA, https://doi.org/10.2210/pdb7tdx/pdb). All study data are included in the article and/or Supplementary data.
