# Supplementary material for: FOXC2 represses NFAT1-dependent transcription through a DNA-facilitated protein–protein interaction
Source: Nucleic Acids Res. 2026 Apr 23;54(8):gkag367. doi: 10.1093/nar/gkag367 (PMC13103735; doi:10.1093/nar/gkag367)
Supplement: gkag367_Supplemental_File [file gkag367_supplemental_file.pdf]

FOXC2 represses NFAT1-dependent transcription through a DNA-facilitated protein–protein interaction

Xiaojuan Chen[1], Sipeng Wu[2], Sitong Yue[3], Lin Zhang[1], Xueru Liu[1], Shuyan Dai[1,4], Jun Li[5], Huajun Zhang[1,6], Hudie Wei[1], Ming Guo[1], Lingzhi Qu[1], Lin Chen[7], Yalan Deng[1,6,*], Yongheng Chen[1,*]

[1] Department of Oncology, NHC Key Laboratory of Cancer Proteomics & State Local Joint Engineering Laboratory for Anticancer Drugs, National Clinical Research Center for Geriatric Disorders, Xiangya Hospital, Central South University, Changsha, Hunan 410008, China

[2] Department of Gastroenterology, Xiangya Hospital, Central South University, Changsha, 410008, Hunan, China

[3] Department of Ultrasound, Xiangyang No. 1 People's Hospital, Hubei University of Medicine, Xiangyang, 441000, China

[4] Department of Pharmacology, Xiangya School of Pharmaceutical Sciences, Central South University, Changsha 410078, China

[5] Institute of Clinical Medicine, The First Affiliated Hospital of University of South China, Hengyang, Hunan, 421001, China

[6] Department of Ultrasonic Imaging, Xiangya Hospital, Central South University, Changsha, 410008, Hunan, China

[7] Molecular and Computational Biology, Department of Biological Sciences, University of Southern California, Los Angeles, CA 90089, USA

[*] To whom correspondence should be addressed. Tel: +86 731 84327542; Fax: +86 731 84327542; Email: yonghenc@163.com

Correspondence may also be addressed to Yalan Deng. Tel: +86 731 84327542; Fax: +86 731 84327542; Email: dengyalan0104@163.com

Supplementary Table S1. Data collection and refinement statistics for NFAT1/FOXC2/DNA complexes.

	NFAT1/FOXC2 /ARRE2	NFAT1/FOXC2 /ARRE2-P	NFAT1/FOXC2 /ARRE3-P
**PDB code**	**9VRQ**	**9VRT**	**9VS2**
**Data collection**			
Wavelength （Å）	0.9793	0.9793	0.9793
Space group	P 21 21 21	P 21 21 21	P 21 21 21
a,b,c(Å)	65.98, 77.34, 117.06	68.06, 79.34, 119.49	67.71, 79.51, 120.57
α, β, γ (°)	90.00, 90.00, 90.00	90.00, 90.00, 90.00	90.00, 90.00, 90.00
Resolution (Å)	38.10-2.80 (2.90-2.80)	39.08-2.65 (2.74-2.65)	47.40-2.65 (2.75-2.65)
Unique reflections	15281 (1483)	19461 (1692)	18641 (1496)
Rsym or Rmerge	0.23 (0.72)	0.40 (1.44)	0.30 (1.19)
Redundancy	12.97 (12.41)	12.6 (12.9)	12.70 (12.6)
Completeness (%)	99.9 (99.8)	97.32 (88.31)	95.46 (77.31)
Mean I/sigma(I)	12.97 (3.44)	5.96 (2.76)	6.84 (2.27)
**Refinement**			
Resolution (Å)	38.10 - 2.80	39.08 - 2.65	47.40 - 2.65
Wilson B-factor	58.09	39.39	45.29
R-work / R-free	0.23/0.26	0.21/0.26	0.21/0.27
Number of non-hydrogens	3913	4001	3962
macromolecules	3875	3902	3891
Protein residues	379	379	378
RMS (bonds)	0.01	0.009	0.004
RMS (angles)	1.26	0.95	0.75
Ramachandran favored (%)	95.68	96.25	96.77
Ramachandran outliers (%)	0.27	0.54	0
Average B-factor	57.20	46.25	48.53
macromolecules	56.20	47.03	49.59
Solvent	44.81	36.02	36.34

Supplementary Table S2. Primer sets for real-time PCR.

Genes		Primer Sequence (5’- 3’)
IL2	Forward	GCACTAAGTCTTGCACTTGTCA
	Reverse	AATGCTCCAGTTGTAGCTGTG
TNF	Forward	AACATCCAACCTTCCCAAACGC
	Reverse	TGGTCTCCAGATTCCAGATGTCAGG
CXCL5	Forward	AGCTGCGTTGCGTTTGTTTAC
	Reverse	TGGCGAACACTTGCAGATTAC
CCL2	Forward	CTCGCTCAGCCAGATGCAAT
	Reverse	TTGGGTTTGCTTGTCCAGGT
GAPDH	Forward	CAAGGCTGAGAACGGGAAG
	Reverse	TGAAGACGCCAGTGGACTC

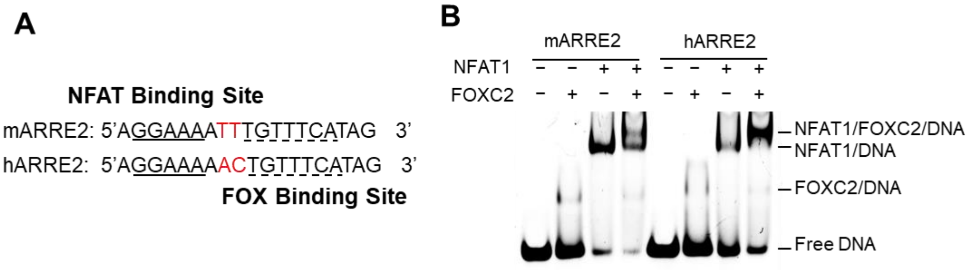

Supplementary Figure S1. Comparative DNA-binding analysis of NFAT1 and FOXC2 with mouse and human ARRE2 elements. (**A**) Sequence alignment of mouse (mARRE2) and human (hARRE2) IL2 promoter elements. Core NFAT and FOX binding motifs are underlined; divergent nucleotides are highlighted in red. (**B**) Binding characteristics of NFAT1 and FOXC2 with mARRE2 and hARRE2 DNAs by EMSA. Protein and DNA were combined at a ratio of 1:1.

A

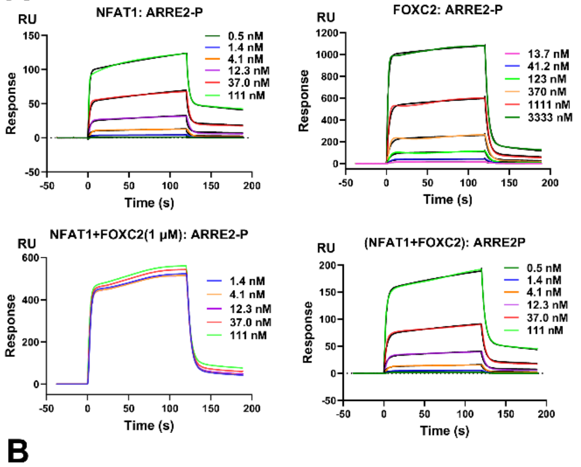

Two state reaction	Immobilized ARRE2-P		
	NFAT1	FOXC2	NFAT1 +FOXC2
ka1 (1/Ms)	$(4.9 \pm 0.8) \times 10^{6}$	$(9.4 \pm 0.9) \times 10^{4}$	$(1.5 \pm 0.1) \times 10^{6}$
kd1 (1/s)	$(5.2 \pm 1.2) \times 10^{-1}$	$(4.1 \pm 0.7) \times 10^{-1}$	$(2.5 \pm 0.0) \times 10^{-1}$
ka2 (1/s)	$(3.2 \pm 1.7) \times 10^{-3}$	$(2.2 \pm 0.4) \times 10^{-3}$	$(3.5 \pm 0.2) \times 10^{-3}$
kd2 (1/s)	$(3.7 \pm 1.3) \times 10^{-3}$	$(8.3 \pm 2.2) \times 10^{-3}$	$(3.6 \pm 0.1) \times 10^{-3}$
**KD (M)**	$\mathbf{(5.4 \pm 0.5) \times 10^{-8}}$	$\mathbf{(3.5 \pm 1.0) \times 10^{-6}}$	$\mathbf{(8.8 \pm 0.7) \times 10^{-8}}$

B

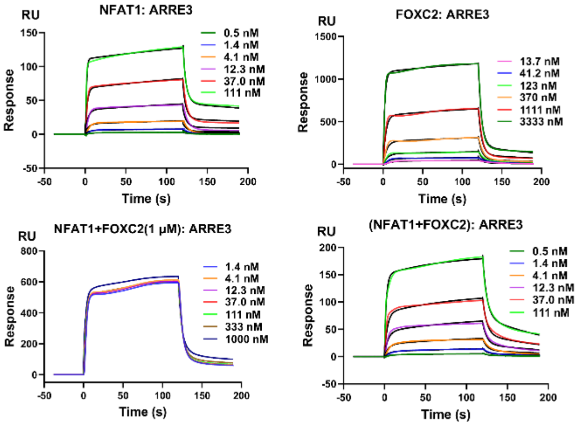

Two state reaction	Immobilized ARRE3		
	NFAT1	FOXC2	NFAT1 +FOXC2
ka1 (1/Ms)	$(2.1 \pm 1.1) \times 10^{7}$	$(8.4 \pm 0.5) \times 10^{4}$	$(2.4 \pm 0.0) \times 10^{6}$
kd1 (1/s)	$(1.3 \pm 0.6) \times 10^{0}$	$(3.1 \pm 0.0) \times 10^{-1}$	$(1.9 \pm 0.0) \times 10^{-1}$
ka2 (1/s)	$(2.3 \pm 1.4) \times 10^{-3}$	$(1.8 \pm 0.0) \times 10^{-3}$	$(5.9 \pm 0.7) \times 10^{-3}$
kd2 (1/s)	$(1.5 \pm 1.0) \times 10^{-3}$	$(5.8 \pm 0.5) \times 10^{-3}$	$(8.8 \pm 0.3) \times 10^{-3}$
**KD (M)**	$\mathbf{(2.0 \pm 0.7) \times 10^{-8}}$	$\mathbf{(2.8 \pm 0.1) \times 10^{-6}}$	$\mathbf{(4.8 \pm 0.0) \times 10^{-8}}$

C

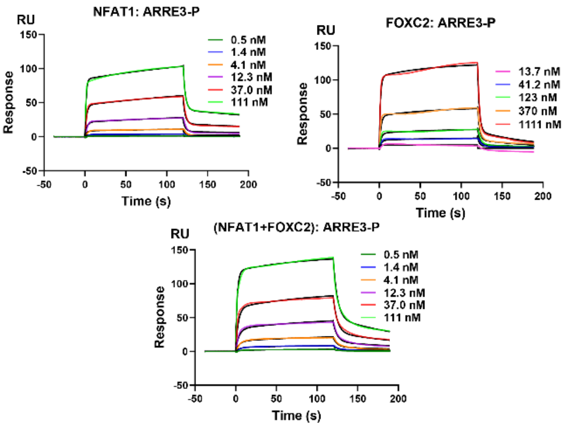

Two state reaction	Immobilized ARRE3-P		
	NFAT1	FOXC2	NFAT1 +FOXC2
ka1 (1/Ms)	$(7.5 \pm 1.2) \times 10^{6}$	$(3.0 \pm 1.0) \times 10^{5}$	$(2.5 \pm 0.3) \times 10^{6}$
kd1 (1/s)	$(6.9 \pm 2.4) \times 10^{-1}$	$(4.8 \pm 0.2) \times 10^{-1}$	$(1.9 \pm 0.0) \times 10^{-1}$
ka2 (1/s)	$(2.5 \pm 1.6) \times 10^{-3}$	$(3.1 \pm 0.4) \times 10^{-3}$	$(5.2 \pm 0.2) \times 10^{-3}$
kd2 (1/s)	$(4.8 \pm 1.8) \times 10^{-3}$	$(1.2 \pm 0.2) \times 10^{-2}$	$(8.1 \pm 0.3) \times 10^{-3}$
**KD (M)**	$\mathbf{(2.0 \pm 0.7) \times 10^{-8}}$	$\mathbf{(1.4 \pm 0.5) \times 10^{-6}}$	$\mathbf{(4.6 \pm 0.7) \times 10^{-8}}$

Supplementary Figure S2. Quantitative SPR analysis of the binding affinities of NFAT1, FOXC2, and the NFAT1–FOXC2 complex to ARRE2-P (**A**), ARRE3 (**B**), and ARRE3-P (**C**) DNA elements. Representative sensor response curves (colored curves) show the binding responses for three-fold serial dilutions of the analytes: NFAT1, FOXC2, pre-incubated NFAT1–FOXC2 mixture, and a complex formed by diluting NFAT1 in the presence of 1 μM FOXC2. Black curves represent the global fit to a two-state reaction model. The derived kinetic and equilibrium constants (mean ± SD, from two independent experiments) are summarized in the table to the right.

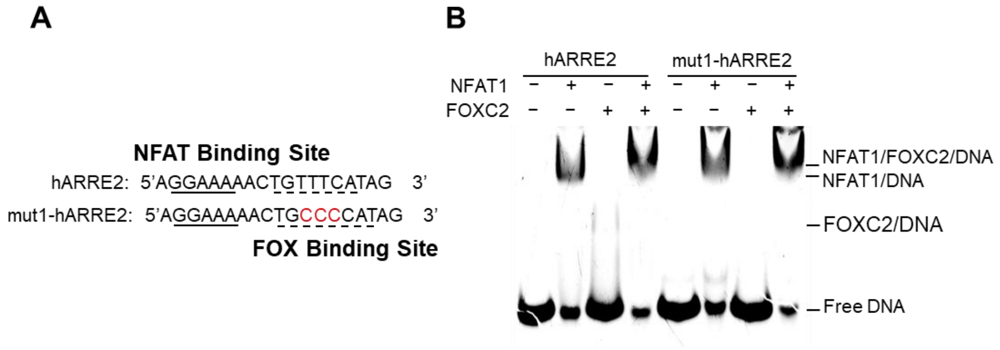

Supplementary Figure S3. DNA binding specificity of NFAT1 and FOXC2 to wild-type and FOX site-mutated hARRE2 elements. (**A**) Sequence comparison of wild-type hARRE2 and its FOX-binding site mutant (mut1-hARRE2), with core binding motifs underlined. (**B**) EMSA demonstrating differential binding of NFAT1 and FOXC2 to wild-type versus mutant hARRE2 at 1:1 molar ratio.

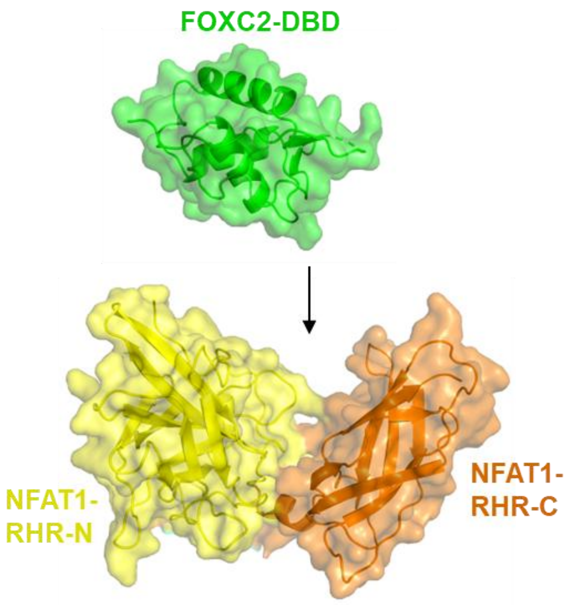

Supplementary Figure S4. Surface representation of FOXC2-DBD inserts into the V-shaped groove of NFAT-RHR.

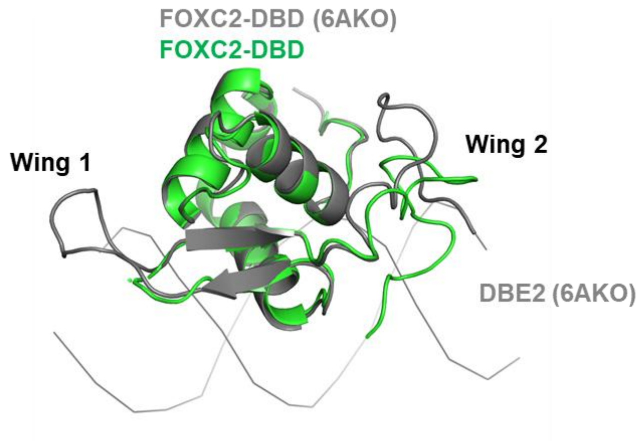

Supplementary Figure S5. Structural comparison of FOXC2-DBD in NFAT1-RHR/FOXC2-DBD/ARRE2 (green) and FOXC2-DBD/DBE2 (PDB: 6AKO) (gray).

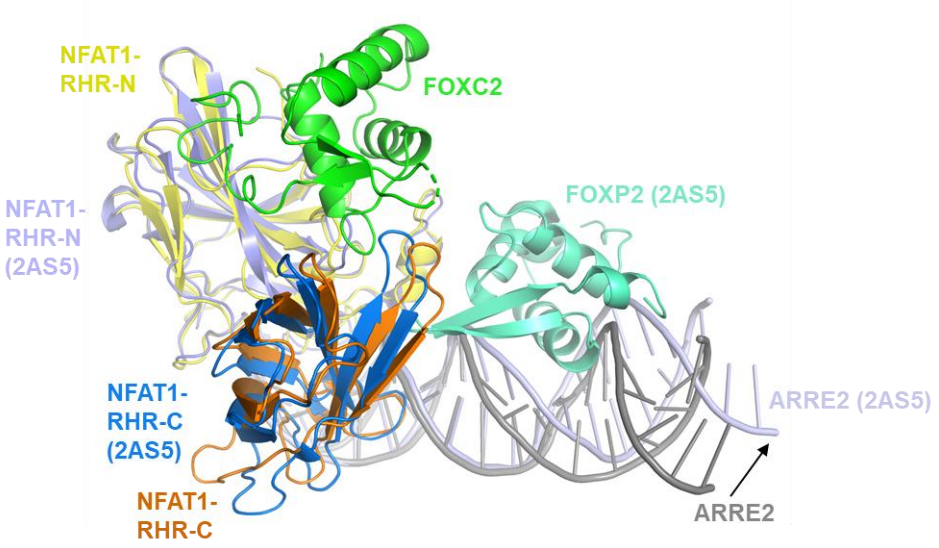

Supplementary Figure S6. Superposition of the complex of NFAT1-RHR/FOXC2/ARRE2 DNA with the previously reported complex of NFAT1-RHR/FOXP2/ARRE2 DNA (PDB: 2AS5).

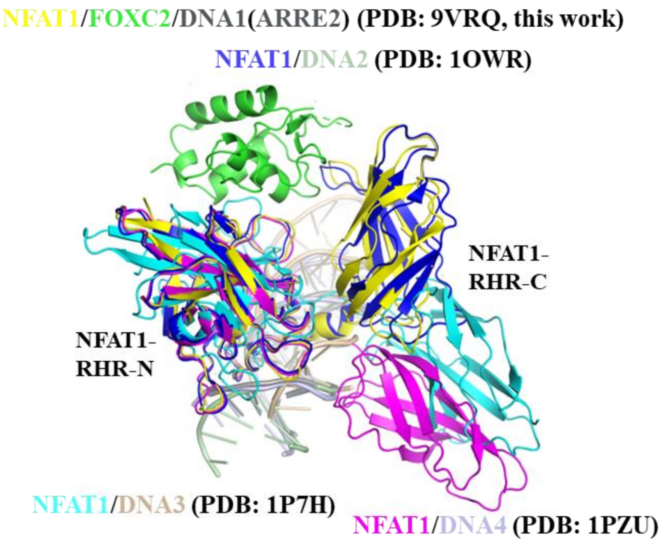

Supplementary Figure S7. Different conformations of NFAT1-RHR-C on binding different DNAs.

FOXC2 to NFAT1-DNA

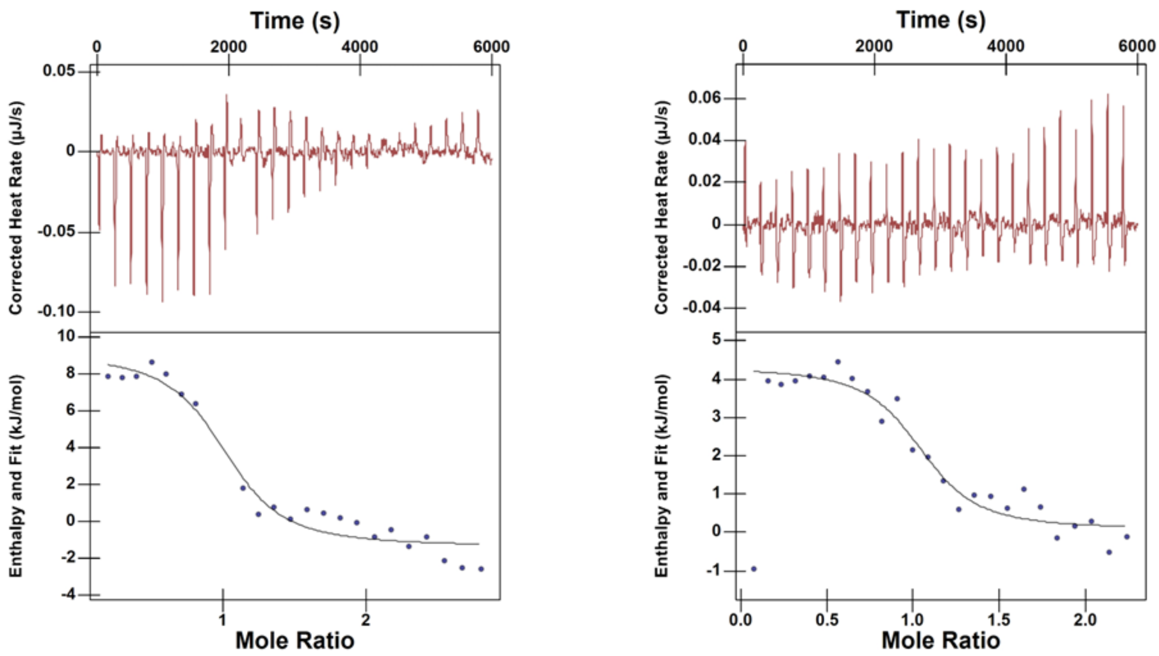

Supplementary Figure S8. ITC heat signals and the integrated curves for FOXC2 binding to NFAT1–DNA complex (1:1 molar ratio) in two replicates.

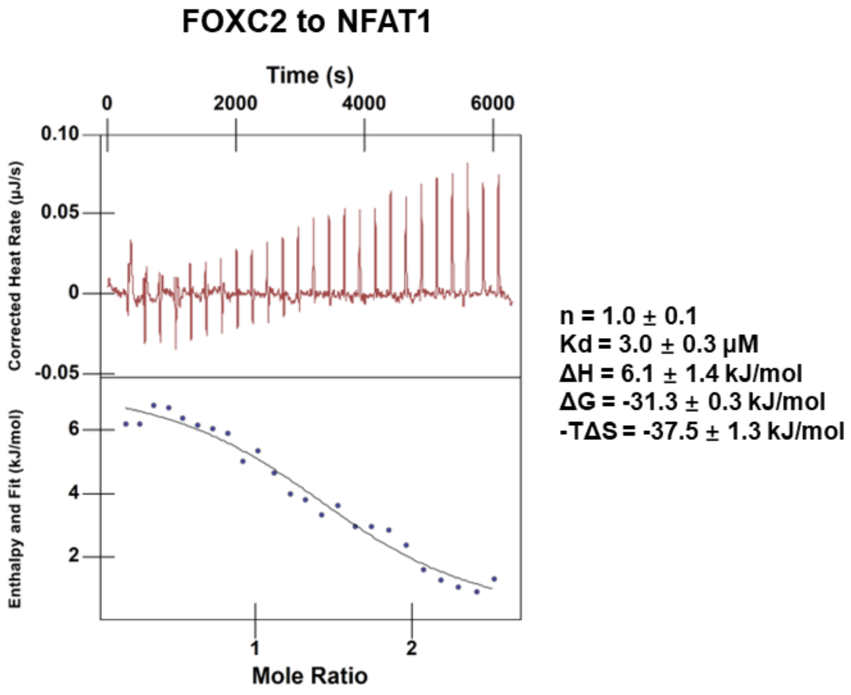

Supplementary Figure S9. Representative thermodynamic analysis of FOXC2–NFAT1 interaction by ITC. The integrated curve subtracted the background heat signals of FOXC2 titration buffer. Data representative of n = 3 independent experiments.

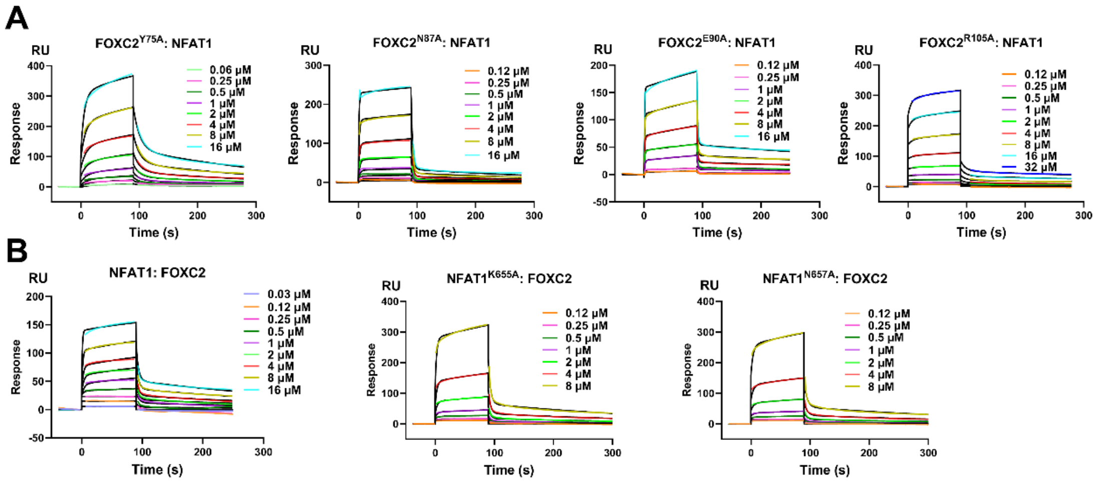

Supplementary Figure S10. Mutational analysis of the NFAT1–FOXC2 interaction by SPR. (**A**) Representative sensor response curve (colored curves) showing the binding of FOXC2 mutants to immobilized NFAT1. (**B**) Representative sensor response curve showing the binding responses of NFAT1 wild-type and mutants to immobilized FOXC2. Black curves represent the global fit to a two-state reaction model.

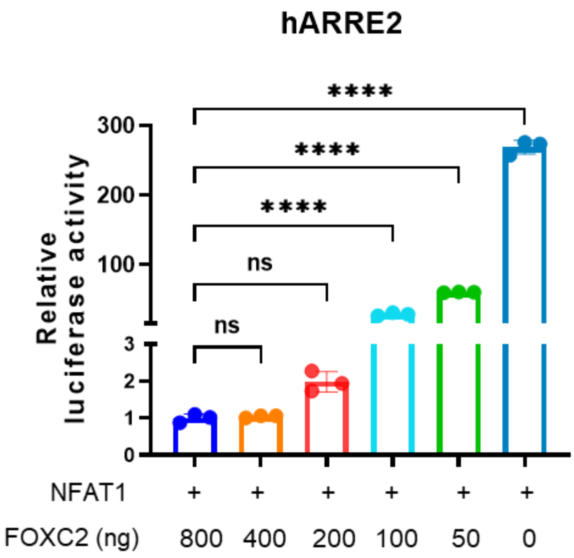

Supplementary Figure S11. Dose-dependent inhibitory effect of FOXC2 on NFAT1 transcriptional activity. HEK293T cells were transfected with an equal amount of the luciferase reporter gene and the NFAT1 plasmid (400 ng), as well as an equal fold increase FOXC2 plasmid. Data are shown the mean ± SD of n = 3 independent replicates. P values were calculated using the transfected 800 ng FOXC2 plasmid group as a control. ** P < 0.01, **** P < 0.0001, ns: not statistically significant (P > 0.05).

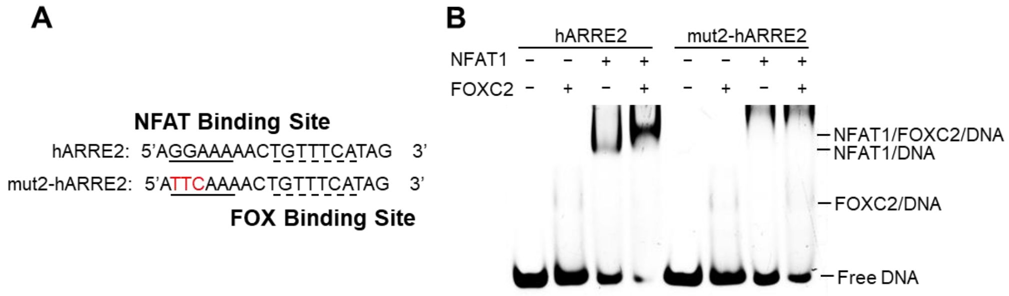

Supplementary Figure S12. DNA binding specificity of NFAT1 and FOXC2 to wild-type and NFAT site-mutated hARRE2 elements. (**A**) Sequence comparison of wild-type hARRE2 and its NFAT-binding site mutant (mut2-hARRE2), with core binding motifs underlined. (**B**) EMSA analysis of the binding effects of FOXC2 and NFAT1 on wild-type and mutated hARRE2 DNA. Protein and DNA were combined at a ratio of 1:1.

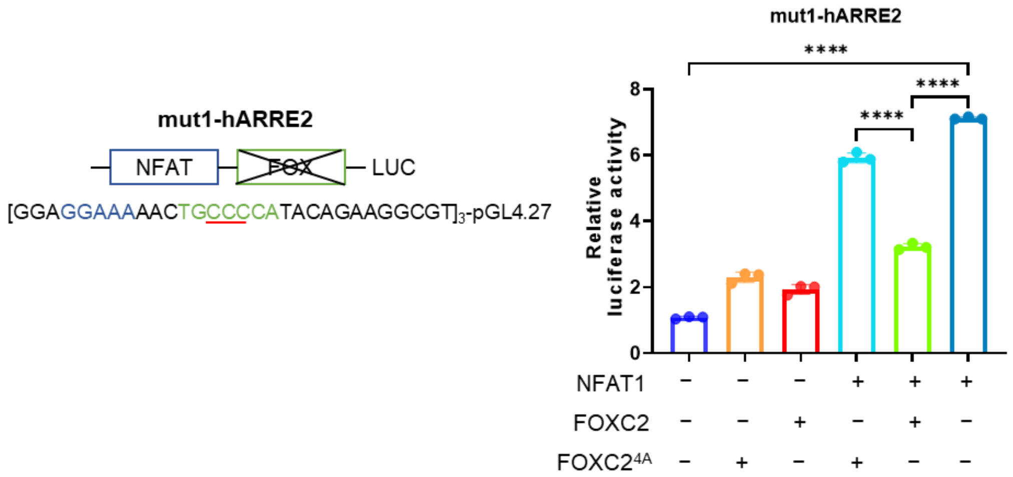

Supplementary Figure S13. FOXC2 still represses the transcriptional activity of NFAT1 as the FOX binding site of the ARRE2 element on the human *IL2* promoter is mutated. HEK293T cells were transfected with equal amounts of luciferase reporter gene plasmid, NFAT1 plasmid, and wild-type or mutant FOXC2 plasmid. Data are shown as mean ± SD (n=3). P values were calculated using empty plasmid group as a control. **** P < 0.0001.

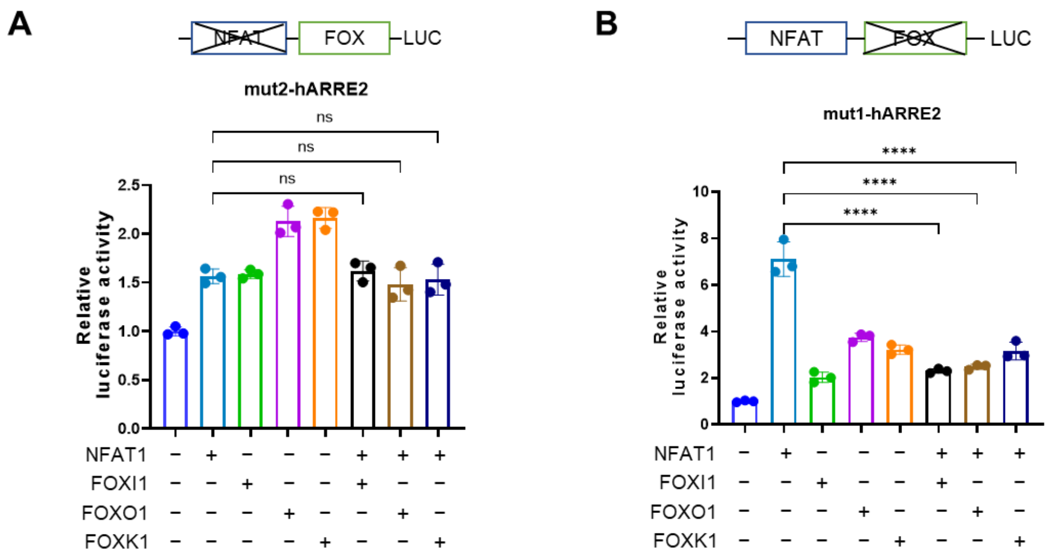

Supplementary Figure S14. Effects of other FOX proteins and NFAT1 on the transcriptional activity of the NFAT1 binding site mutated (**A**) or FOX binding site mutated (**B**) ARRE2 element. HEK293T cells were transfected with equal amounts of mutant luciferase reporter gene plasmids, NFAT1 plasmid, and FOX plasmid. Data are shown the mean ± SD (n=3). P values were calculated using empty plasmid group as a control. **** P < 0.0001, ns: not statistically significant (P > 0.05).

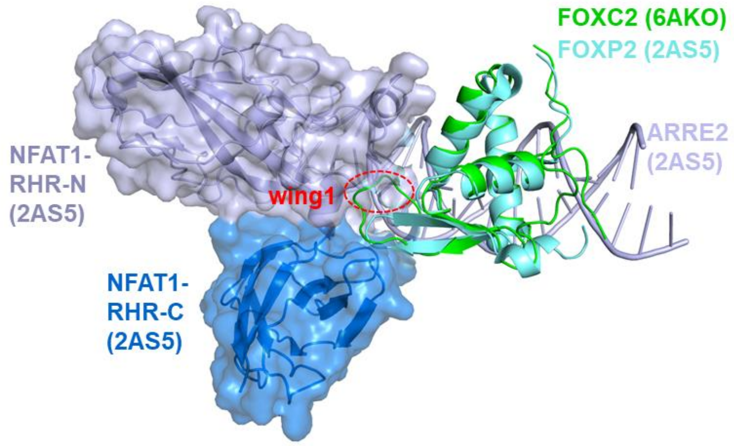

Supplementary Figure S15. Structural superposition suggests a wing1-driven steric clash with NFAT1. This model was generated by structural superposition of the FOX binding sites from the NFAT/FOXP2/ARRE2 (PDB: 2AS5) and FOXC2/DBE2 (PDB: 6AKO) complexes.

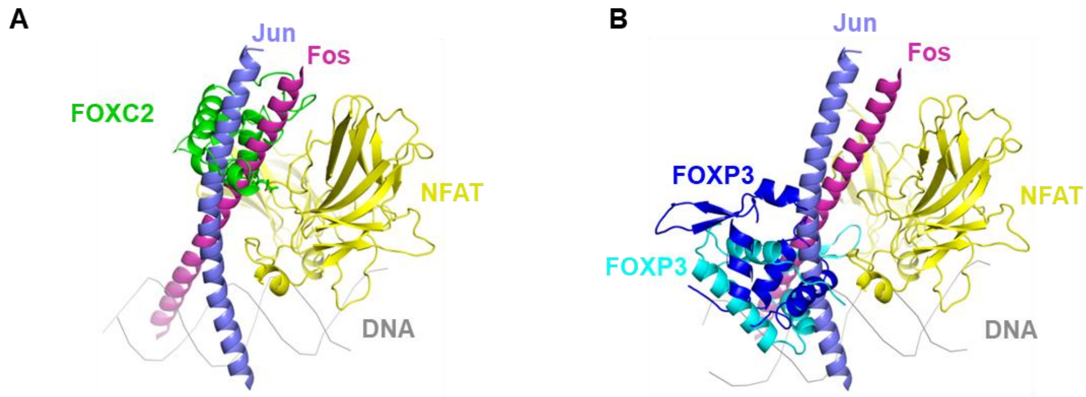

Supplementary Figure S16. FOX proteins and AP-1 (the Jun/Fos complex) are predicted to compete for overlapping binding sites in forming NFAT-associated transcriptional complexes. (**A**) Superposition of the NFAT/FOXC2/DNA complex with the previously reported NFAT/AP1/DNA (PDB: 1A02) complex. (**B**) Superposition of the NFAT/AP1/DNA (PDB: 1A02) complex with NFAT/FOXP3/DNA (PDB: 3QRF) complex.
